# The active microbial community more accurately reflects the anaerobic digestion process: 16S rRNA (gene) sequencing as a predictive tool

**DOI:** 10.1186/s40168-018-0449-9

**Published:** 2018-04-02

**Authors:** Jo De Vrieze, Ameet J. Pinto, William T. Sloan, Umer Zeeshan Ijaz

**Affiliations:** 1 Center for Microbial Ecology and Technology (CMET), Ghent University, Coupure Links 653, B-9000 Ghent, Belgium; 2 Infrastructure and Environment Research Division, School of Engineering, University of Glasgow, Rankine Building, Oakfield Avenue, Glasgow, G12 8LT UK; 3 Northeastern University, 360 Huntington Avenue, Boston, MA 02115 USA

**Keywords:** Biogas, Illumina sequencing, Methane, Methanogenesis

## Abstract

**Background:**

Amplicon sequencing methods targeting the 16S rRNA gene have been used extensively to investigate microbial community composition and dynamics in anaerobic digestion. These methods successfully characterize amplicons but do not distinguish micro-organisms that are actually responsible for the process. In this research, the archaeal and bacterial community of 48 full-scale anaerobic digestion plants were evaluated on DNA (total community) and RNA (active community) level via 16S rRNA (gene) amplicon sequencing.

**Results:**

A significantly higher diversity on DNA compared with the RNA level was observed for archaea, but not for bacteria. Beta diversity analysis showed a significant difference in community composition between the DNA and RNA of both bacteria and archaea. This related with 25.5 and 42.3% of total OTUs for bacteria and archaea, respectively, that showed a significant difference in their DNA and RNA profiles. Similar operational parameters affected the bacterial and archaeal community, yet the differentiating effect between DNA and RNA was much stronger for archaea. Co-occurrence networks and functional prediction profiling confirmed the clear differentiation between DNA and RNA profiles.

**Conclusions:**

In conclusion, a clear difference in active (RNA) and total (DNA) community profiles was observed, implying the need for a combined approach to estimate community stability in anaerobic digestion.

**Electronic supplementary material:**

The online version of this article (10.1186/s40168-018-0449-9) contains supplementary material, which is available to authorized users.

## Background

Anaerobic digestion (AD) relies on complex microbial communities for the conversion of organic waste streams into biogas. The application of online monitoring strategies via conventional operational parameters, such as pH, volatile fatty acid (VFA) concentration, gas composition and alkalinity [1–4], resulted in the expansion of high-rate AD systems to industrial scales. These physico-chemical parameters reflect the current state of the process and do not accurately reflect the microbial community composition, dynamics, or activity [5, 6], nor do they allow future process performance prediction. Attempts to relate molecular and operational parameters have resulted in significantly different outcomes with respect to the relation between diversity and process performance [7–10]. Hence, we are to implement microbial process control of AD or microbial community function in general [11], as also postulated in the microbial resource management concept [12–15]. Then, we need to extend our knowledge of the interaction between the temporal trajectories of microbial community structure and operational parameters.

The advent of high-throughput sequencing techniques in AD research resulted in a significant increase in our understanding of the (active) microbial community [16]. Amplicon sequencing helped to identify the *Actinobacteria*, *Bacteroidetes*, *Chloroflexi*, *Firmicutes* and *Proteobacteria* as dominant bacterial phyla and helped reveal several acetoclastic and hydrogenotrophic methanogens [17–19]. Acetoclastic *Methanosaeta* mainly have been observed at stable process conditions [20], while a transition to hydrogenotrophic methanogenesis often took place at deteriorating conditions, related to an increase in salinity, total ammonia nitrogen (TAN) concentration, or other compounds that negatively affect *Methanosaeta* [21]. Application of ‘omics’ techniques has helped elucidate the important genes involved in carbohydrate, lipid and protein metabolism in AD [22–26]. Carbon isotope analysis methods made the determination of the dominant methanogenic pathway possible [27, 28] and clarified whether or not it is coupled with syntrophic acetate oxidation [29]. The combination of carbon-based stable isotope probing coupled with amplicon has helped identify microorganisms involved with specific pathways in the process of methanogenesis [30, 31].

The DNA-based techniques have delivered significant insights, but they do exhibit important shortcomings in their ability to reveal the active microbial community in AD. The ‘omics’ techniques suffer from two main issues. First, reference databases (although this is only an issue for reference-based assembly, not for de novo assembly) are often incomplete [32], which leads to a limited degree of reads assignment [33], which results in a substantial lack of data interpretation. Second, often contradictory results are obtained when comparing different ‘omics’ techniques [23, 34] or when comparing ‘omics’ techniques with alternative methods [33]. Carbon isotope-based methods are restricted by the fact that the metabolic pathway and/or the micro-organisms involved in the degradation of only a single and known substrate can be monitored. Collaboration between micro-organisms is a crucial aspect in AD, which is not addressed by any of the abovementioned techniques. Hence, an alternative approach is needed to bridge the knowledge gap on active microbial communities, (potential) collaboration and complete functionality prediction.

In this research, the microbial community in full-scale AD plants was evaluated through amplicon sequencing of the 16S rRNA gene and the 16S rRNA transcripts to directly compare the total and active microbial community. This is in contrast to most other approaches that make use of different techniques to make an estimation of the difference between the active and total microbial communities. The bacterial and archaeal (methanogenic) differential abundance and activity patterns were identified and related to the sensitivity of the methanogenic community to variations in operational parameters in AD. Co-occurrence patterns were created to estimate the difference between mere occurrence and activity to propose potential collaborative associations. Functional prediction profiles were generated to relate potential differences in the DNA and RNA profiles with predicted functionality.

## Methods

### Sample and data collection

Digestate samples were collected from 48 full-scale AD plants in Belgium in 1-L air-tight containers and transported to the laboratory immediately. Upon arrival in the laboratory, samples were homogenized and three replicate 1.5 mL subsamples were taken and stored at − 80 °C until DNA and RNA extraction. Another 10 mL subsample was stored at − 20 °C for VFA analysis. A 50-mL sample was stored at 4 °C for TAN, conductivity, volatile solids (VS), total solids (TS) and cation analysis. Sample pH was measured directly upon arrival in the laboratory. Information concerning the sludge retention time (SRT) and temperature were obtained directly from the operator.

### Simultaneous DNA and RNA extraction

Total DNA and RNA were co-extracted from the same sample to avoid biases related to variable cell lysis efficiency. The RNA PowerSoil® Total RNA Isolation Kit in combination with the RNA PowerSoil® DNA Elution Accessory Kit (Mobio Laboratories Inc., Carlsbad, CA, USA) was used for simultaneous RNA and DNA extraction. Samples were transferred immediately from the − 80 °C freezer to liquid N_2_ to prevent RNA from degrading during thawing. Next, 1.0 g of frozen digestate sample was transferred to the Bead Tubes, which were also maintained in liquid nitrogen. After removing the Bead Tubes with samples from the liquid N_2_, buffer solutions (Bead Solution and solutions SR1 and SR2) were added immediately to minimize RNA degradation. The remaining steps in the protocol were identical to the recommendations of the manufacturer.

The RNA extracts were subjected to DNase treatment using the DNase I Kit for Purified RNA in Solution (Mobio Laboratories Inc.) for removal of residual DNA. Efficiency of DNA removal was tested according to Boon et al. [35], which involved PCR amplification of the bacterial 16S rRNA genes using primers P338f and P518r [36], followed by visualization of the PCR production on 1% agarose gel electrophoresis to confirm the absence of DNA. The RNA was subsequently converted to cDNA using the qScriber™ cDNA Synthesis Kit (Mobio Laboratories Inc.). The final quality of the cDNA and DNA was validated by 1% agarose gel electrophoresis and PCR analysis, as described earlier.

### Amplicon sequencing and data processing

The cDNA and DNA extracts were sent to LGC Genomics GmbH (Berlin, Germany) for sequencing on the Illumina Miseq platform. Sequencing was performed by targeting the V3-V4 hypervariable region of the 16S rRNA (gene) using bacterial primers 341F and 785R (Additional file 1: Table S1) [37], with an additional wobble position in the reverse primer to make it more universal. A nested approach was used for the archaea, with the archaea specific primers 340F and 1000R for the first PCR run [38], followed by universal primers 341F and 806R [18] for the second PCR run (Additional file 1: Table S1). The PCR protocol was carried out as described in SI (S1). The PCR products were pooled and purified with Agencourt AMPure XP beads (Beckman Coulter, Brea, CA). An additional purification was carried out using the MinElute PCR Purification Kit (Qiagen, Venlo, The Netherlands). The purified amplicon pools were used to generate Illumina compatible libraries by adaptor ligation, using the Ovation Rapid DR Multiplex System 1-96 (NuGEN, San Carlos, CA). Illumina compatible libraries were pooled, and size was selected by preparative gel electrophoresis.

Amplicon sequences were trimmed and quality-filtered using Sickle v1.200 [39] with a sliding window approach, removing reads with an average quality score below 20. The BayesHammer error correction tool [40] coupled with the Spades v2.5.0 assembler was used for error correction of the paired-end reads. PANDAseq v2.4 [41] was applied to assemble the paired-end reads, using a minimum overlap of 20. These three steps reduce the substitution error rates significantly (~ 90%) [42]. The UPARSE (v7.0.1001) pipeline [43] was used for operational taxonomic unit (OTU) construction. Briefly, the reads were dereplicated and sorted by decreasing abundance, and singletons were discarded after which simultaneous chimera filtering and OTU clustering was performed, based on 97% similarity. An additional chimera removal step was carried out by means of a reference-based chimera filtering step using the ‘gold’ database (http://drive5.com/uchime/gold.fa), derived from the ChimeraSlayer reference database in the Broad Microbiome Utilities (http://microbiomeutil.sourceforge.net). Representative sequences from OTUs were taxonomically classified against the Ribosomal Database Project (RDP) database, using the standalone RDP Classifier v2.6 [44]. Phylogenetic distances between OTUs were determined using MAFFT v7.040 [45], followed by the construction of an approximately maximum-likelihood phylogenetic tree by means of FastTree v2.1.7 [46]. Functional profiles were predicted based on the 16S rRNA data using Tax4Fun [47] by blasting the OTUs against the SILVA seed v115 and KEGG database release 64.0. After normalizing the data for 16S rRNA gene copy numbers, using the copy numbers obtained from the NCBI genome annotations [47], functional profiles were generated by means of the ultrafast protein classification (UProC) tool [48].

### Statistical analysis

A table with the abundance of OTUs and their taxonomic assignments in each sample was generated (Additional files 2 and 3). Statistical analyses were performed in R Studio version 3.2.3. (http://www.r-project.org) [49] using the packages vegan [50] and phyloseq [51] for community analysis. Heatmaps on different phylogenetic levels were constructed using the *pheatmap* function (pheatmap package). Diversity parameters of the DNA and RNA community profiles were compared using analysis of variance (ANOVA, *aov* function). Non-metric distance scaling (NMDS) plots of community data were generated using the Bray-Curtis, weighted and unweighted UniFrac distance measures. Multivariate homogeneity of dispersion (variance) between DNA and RNA profiles were calculated using the *betadisper* function (vegan), a multivariate analogue of Levene’s test for homogeneity of variances. The original distance matrices were reduced to principal coordinates after which ANOVA was performed. This information was also used to determine the phylogenetic distance between the DNA and the RNA profiles of each sample using ANOVA and linear models (*lm* function). Permutational ANOVA (PERMANOVA) was carried out to evaluate the effect of operational parameters on both DNA and RNA profiles using the *adonis* function (vegan), and the significant parameters were used for canonical correspondence analysis (CCA) plotting. The OTUs that showed a significant difference in terms of DNA and RNA were identified with the *DESeqDataSetFromMatrix* function from the DESeq2 package [52], and correlations with operational data were determined with the Kendall rank coefficient correlation with *P* values adjusted for multiple comparisons using the Benjamini-Hochberg correction [53]. Co-occurrence networks construction and subcommunity analysis were carried out based on the recommendations of Williams et al. [54]. The samples were rarefied, followed by estimation of the Spearman’s rank correlation between each pair of OTUs. The *P* values were corrected for multiple comparisons using the Benjamini-Hochberg correction. Only OTU pairs with a corrected highly significant (*P* < 0.001) correlation were incorporated in the co-occurrence networks. Subcommunities of OTUs with a correlation coefficient > 0.5 were identified [55]. The network statistics Degree, Betweenness, Closeness and Eigenvector centrality were calculated to identify potential keystone species based on their central role in the co-occurrence network [56, 57]. Co-occurrence networks were constructed with the igraph (http://igraph.org), sna [58] and network [59] packages. The KEGG Orthology (K) numbers that showed a significant difference between the DNA and RNA microbial community profiles were identified with the Kruskal-Wallis rank sum test with Benjamini-Hochberg correction. The pathways were visualized with the pathview package [60].

### Analytical techniques

Total solids (TS), volatile solids (VS) and TAN were determined according to standard methods [61]. The pH and conductivity were measured with a C532 pH and C833 conductivity meter (Consort, Turnhout, Belgium), respectively. The free ammonia (NH_3_) concentration was calculated based on the TAN concentration, pH and temperature in the digester. The concentrations of the cations Na^+^, K^+^, Ca^2+^ and Mg^2+^ were determined via ion chromatography (IC, Metrohm IC 761, Herisau, Switzerland) with a Metrosep C6 e 250/4 column and Metrosep C4 Guard/4.0 guard column. The eluent contained 1.7 mM HNO_3_ and 1.7 mM dipicolinic acid. Sample preparation was carried out by centrifugation at 10,000*g* for 10 min, followed by filtration over a 0.45-μm filter (type PA-45/25, Macherey-Nagel, Germany) to remove all solids, and dilution with milli-Q water. The concentrations of the different VFA were analysed by means of gas chromatography, as described in SI (S2).

## Results

### Full-scale digester operational characteristics

Operational data and samples were collected from both mesophilic and thermophilic continuous stirred tank reactor (CSTR) and dry anaerobic composting (DRANCO) AD plants at a specific time point during a period of constant biogas production. The operational parameters differed considerably between the different digesters (Additional file 1: Table S2). The pH ranged between 7.3 and 8.5, while TAN and free ammonia concentrations showed values between 605 and 5971 and 37 and 1585 mg N L^−1^. The overall salinity was represented by the conductivity, with values between 10.3 and 64.6 mS cm^−1^, which mainly related to the Na^+^ (0.1–8.8 g L^−1^), K^+^ (0.6–6.9 g L^−1^) and TAN concentrations, as Ca^2+^ and Mg^2+^ concentrations did not exceed 0.5 and 0.2 g L^−1^, respectively. Total VFA concentration ranged between 0.08 and 27.5 g COD L^−1^ and mainly consisted of acetate (57.1 ± 31.4%) and propionate (28.8 ± 28.8%).

### Microbial composition and diversity in the total (DNA) and active (RNA) community

Separate amplicon sequence data analysis of the bacterial and archaeal community (both DNA and RNA samples) resulted in 795 and 137 OTUs, respectively, and an average of 4242 ± 1791 reads for bacteria and 6427 ± 4950 reads for archaea.

**Fig. 1:**
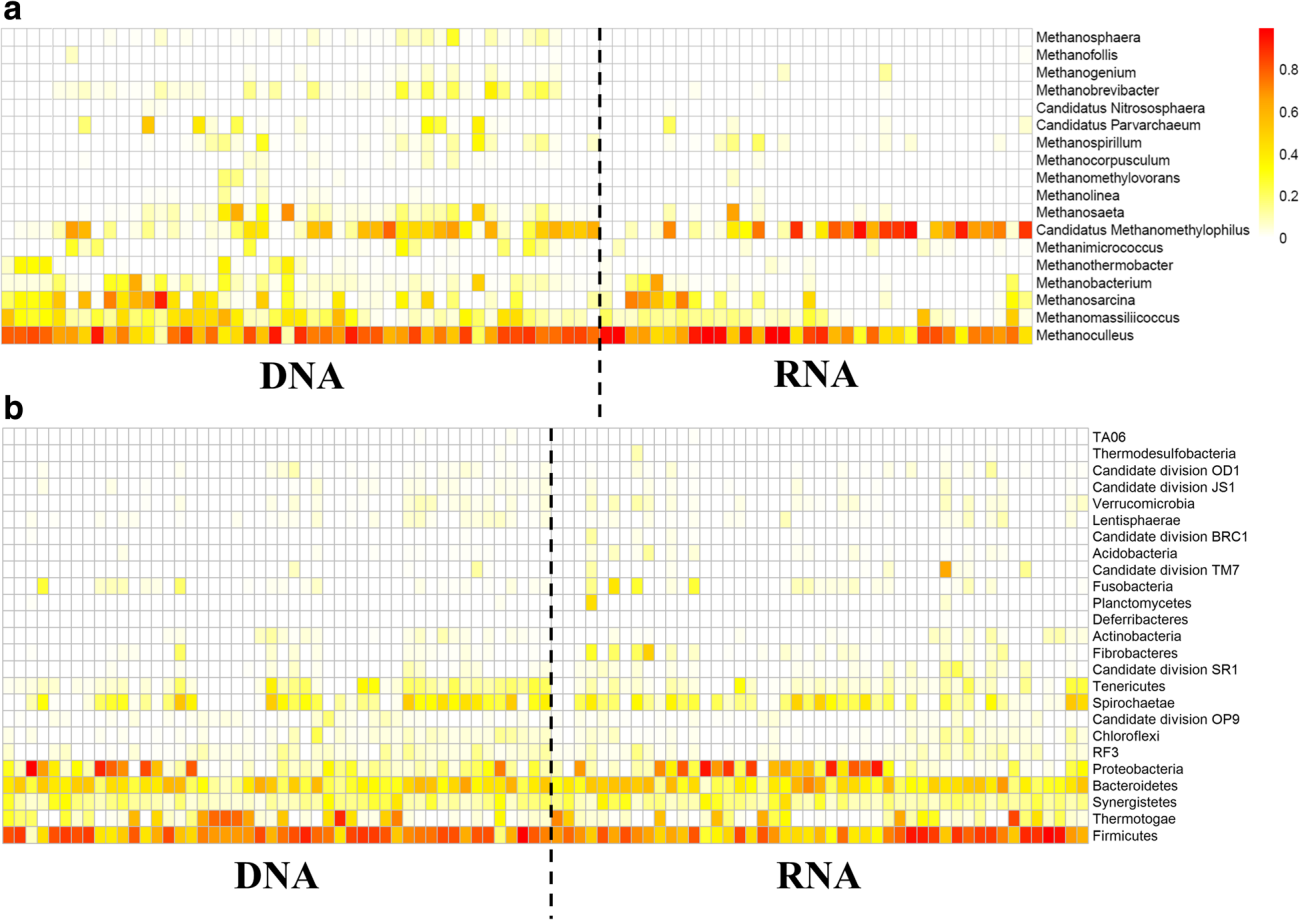
Heatmap representing the square root transformed relative abundance of **a** the archaeal genera and **b** the bacterial phyla of the DNA and RNA profiles of the different samples. The colour scale ranges from 0 (white) to 100% (red) relative abundance

The archaeal community contained OTUs classified as acetoclastic and hydrogenotrophic methanogens, with 20 OTUs classified as Candidatus *Methanomethylophilus*, 14 as *Methanoculleus*, 10 as *Methanosaeta*, 8 as *Methanobrevibacter* and 7 as *Methanosarcina*. An overall dominance of *Methanoculleus* (52.2 ± 26.0%) and Candidatus *Methanomethylophilus* (22.2 ± 26.7%) was observed, while *Methanosaeta* was much less abundant (2.6 ± 8.9%) (Fig. 1a). In total, 58 archaeal OTUs (42.3%), both acetoclastic and hydrogenotrophic methanogens, showed a significant (*P* < 0.05) difference in relative abundance between DNA- and RNA-derived data (Additional file 1: Figure S1). For 17 OTUs, a higher relative abundance was observed on RNA level, which included 3 OTUs classified as *Methanosarcina*. The bacterial community mainly contained OTUs in the *Firmicutes* (45.7 ± 21.8%), *Bacteroidetes* (16.4 ± 10.8%), *Proteobacteria* (15.5 ± 24.6%), *Thermotogae* (9.3 ± 17.2%) and *Spirochaetae* (5.5 ± 7.1%) phyla (Fig. 1b). A significant difference (*P* < 0.05) between DNA and RNA profiles was observed for 203 bacterial OTUs (25.5%) of which 121 and 82 were significantly higher in relative abundance at the DNA level and RNA level, respectively (Additional file 1: Figure S2). The 17 OTUs classified in the *Pseudomonas* genus had a significantly higher relative abundance in RNA, as compared with the DNA data, which was also the case for the 4 OTUs classified as *Tepidanaerobacter* genus. In contrast, the 16 OTUs classified as *Fastidiosipila* genus, the 10 OTUs classified as *Caldicoprobacter* genus, the 7 OTUs classified as *Caldilineaceae* family and the 3 OTUs classified as *Syntrophaceticus schinkii* showed a significantly higher relative abundance at the DNA level as compared with the RNA level.

**Fig. 2:**
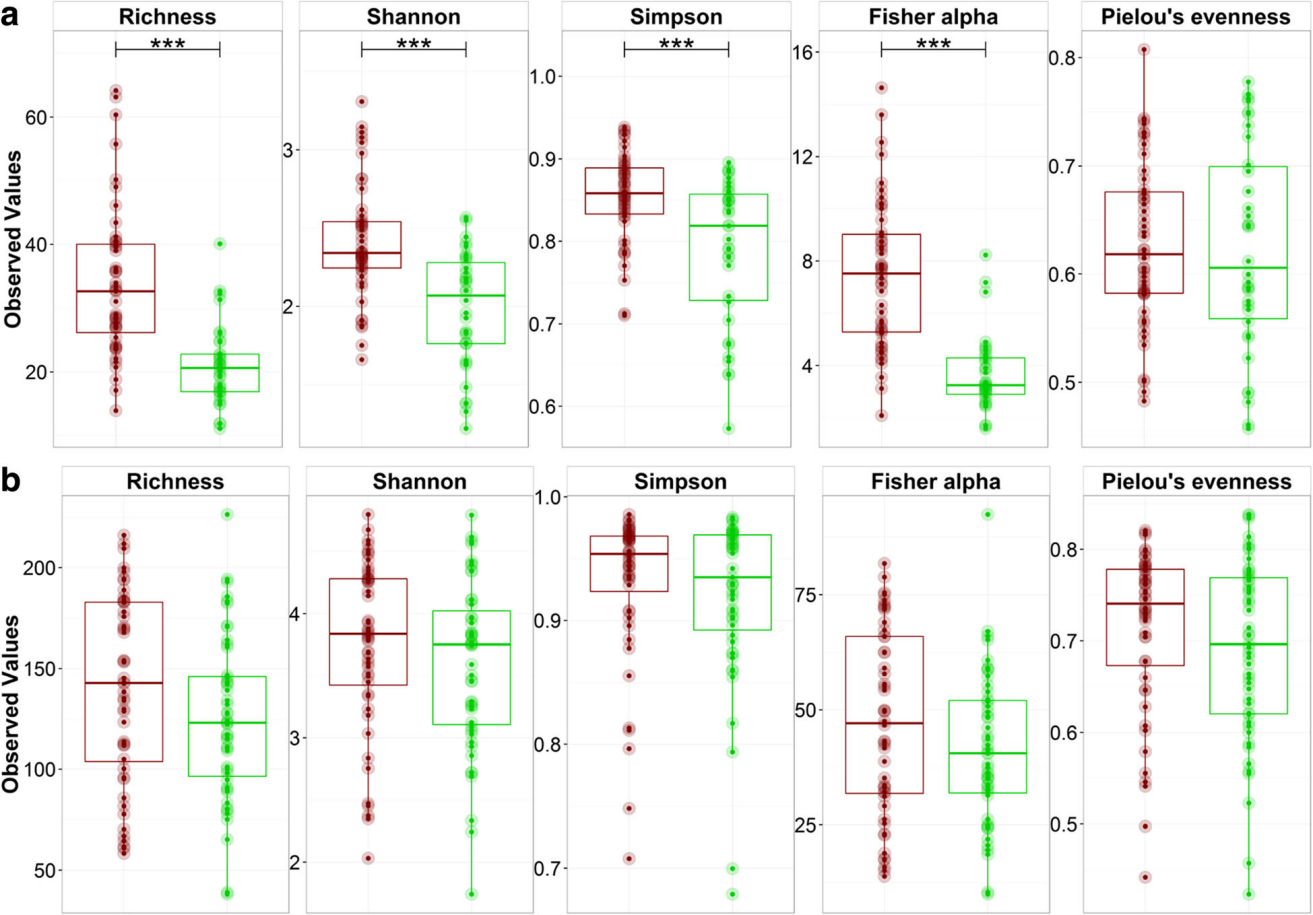
Boxplots of the alpha diversity indices of the **a** archaeal and **b** bacterial community on DNA (red) and RNA (green) level. Significant differences between DNA and RNA are indicated (***)

Basic alpha diversity analysis showed a significantly higher richness (*P* < 0.0001) and overall diversity (*P* < 0.0001), based on the Shannon, Simpson and Fisher alpha, for DNA-based data as compared with the RNA data for archaea, while Pielou’s evenness was similar at both levels (Fig. 2). In contrast, none of the diversity indices showed a significant difference (*P* > 0.05) between DNA and RNA for bacteria.

**Fig. 3:**
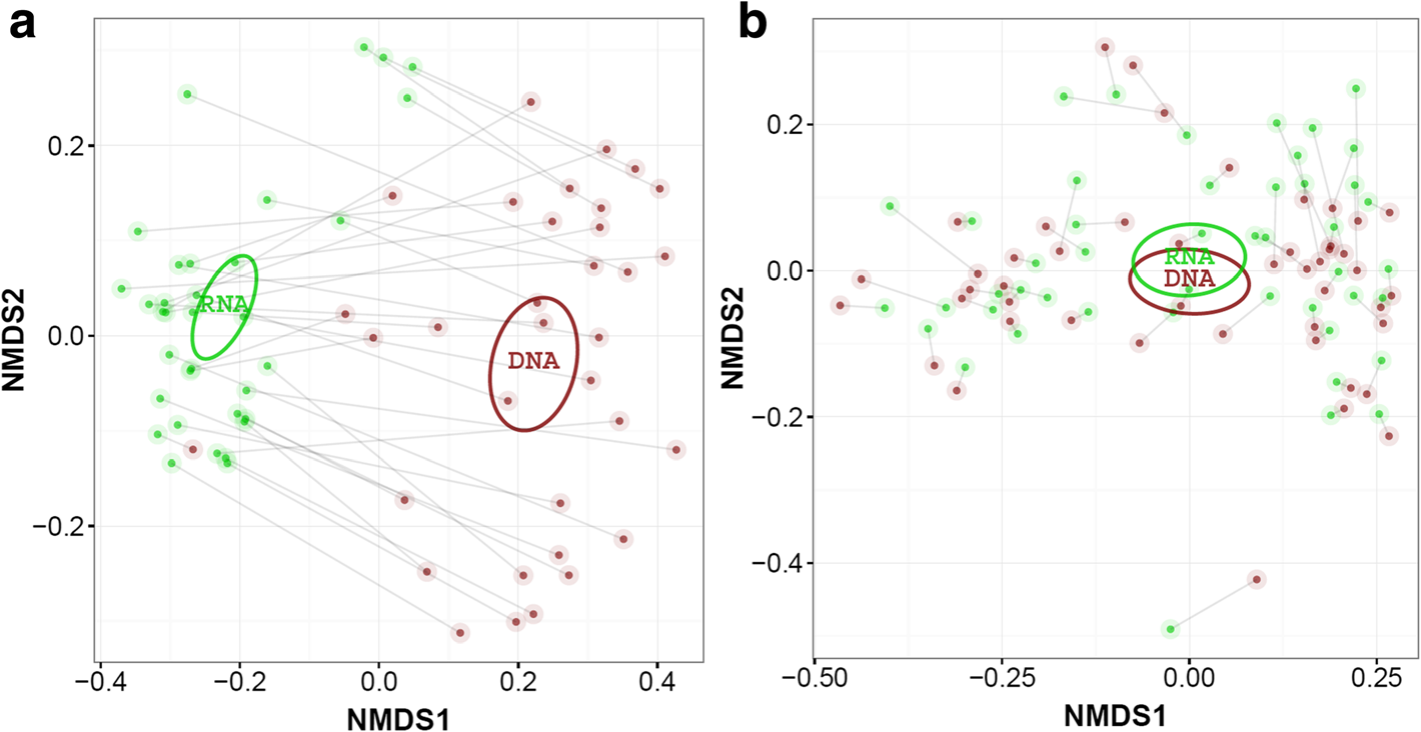
Non-metric distance scaling (NMDS) analysis of the unweighted UniFrac distance indices of the **a** archaeal and **b** bacterial community at OTU level. The DNA (red)-and RNA (green)-based community profiles of the same samples were connected by means of a grey line. The circles represent the 95% value of the standard error of the average value of the DNA (red) and RNA (green) indices

Beta diversity analysis revealed a highly significant (*P* = 0.0001) differentiation pattern between DNA and RNA for archaea using the unweighted UniFrac distance measure (Fig. 3), which calculates distances between samples based on phylogenetic relatedness of the observed OTUs in the samples without taking into account their abundance. This difference was less pronounced for bacteria, with a significant (*P* = 0.025) difference between DNA and RNA. Bray-Curtis dissimilarity and weighted UniFrac (phylogenetic relatedness weighted by abundance) distance measures showed a highly significant difference between DNA and RNA, both for archaea (*P* = 0.0003 and 0.0059, respectively) and bacteria (*P* = 0.0001 and 0.0001, respectively) (Additional file 1: Figure S3). A significantly (*P* < 0.0001) higher degree of variance was observed for DNA-based data for archaea compared with the RNA data using the unweighted UniFrac distance metric, while the opposite was true based on the weighted UniFrac analysis (*P* = 0.023), though less pronounced (Additional file 1: Figure S4).

### Associations between operational conditions on the total and active microbial community

**Fig. 4:**
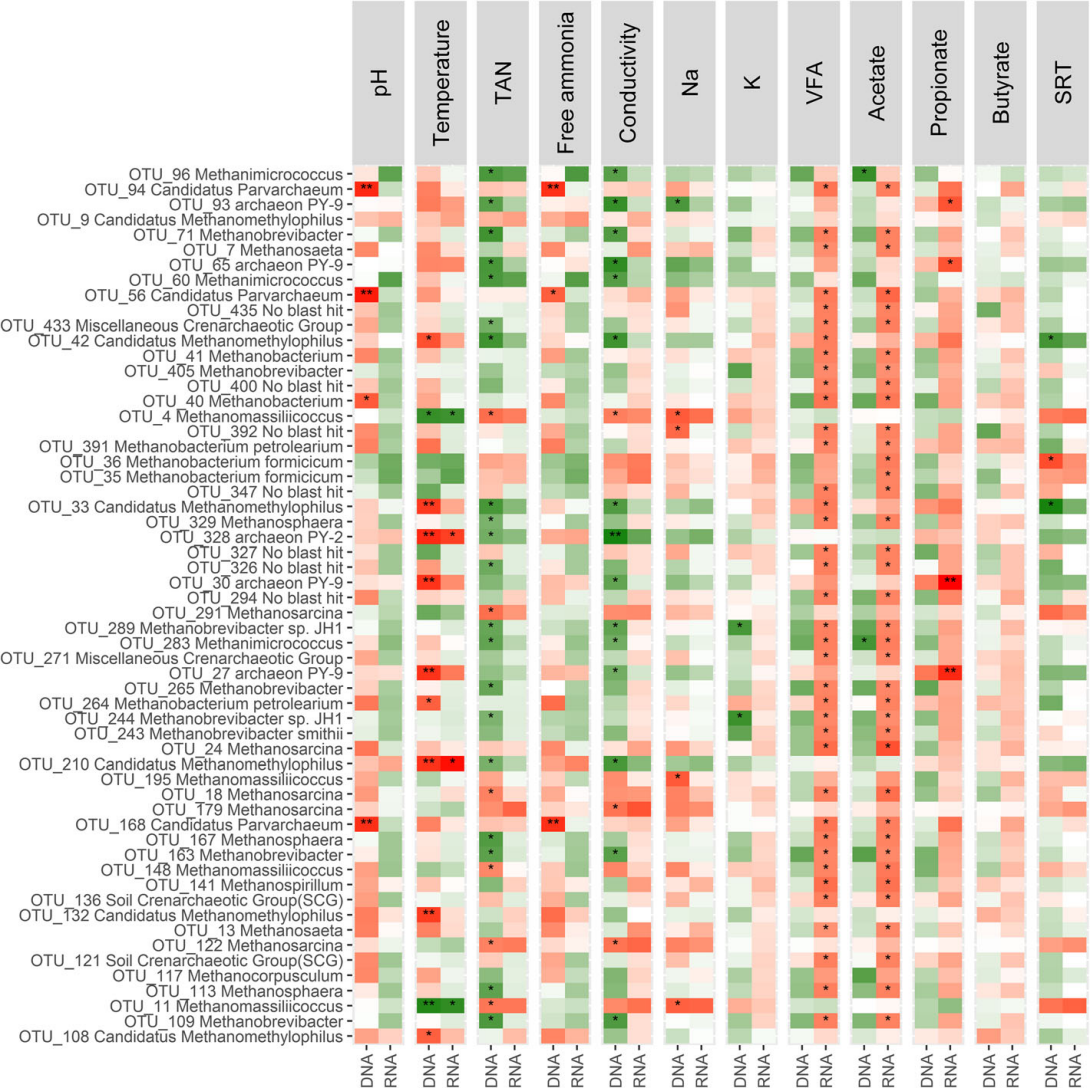
Correlation pattern of the 58 archaeal OTUs that had a significant (*P* < 0.05) difference in relative abundance between DNA and RNA with the main operational parameters, based on the Kendall rank correlation coefficient. Significant positive (green) or negative (red) correlations are marked with * (*Adj. P* < 0.05), ** (*Adj. P* < 0.01), or *** (*Adj. P* < 0.001). TAN total ammonia nitrogen, VFA volatile fatty acids, SRT sludge retention time

The archaeal and bacterial OTUs that were identified as being significantly different in relative abundance between DNA and RNA were evaluated for their correlation with operational parameters. From the 58 archaeal OTUs with a significant difference in relative abundance between DNA and RNA, 38 archaeal OTUs significantly (*P* < 0.05) correlated with total VFA and acetate at the RNA level, while on only two OTUs showed a significant positive correlation with acetate at the DNA level (Fig. 4). Several OTUs also showed a significant correlation with TAN (20 positive and 6 negative) and conductivity (15 positive and 3 negative) at the DNA level, while no significant correlations were present at the RNA level. All OTUs with a significant positive correlation with TAN and conductivity at the DNA level were hydrogenotrophic methanogens, while the OTUs with a significant negative correlation with total VFA and acetate were both acetoclastic and hydrogenotrophic methanogens.

Both significant positive and negative correlations were present for the 203 bacterial OTUs that exhibited a significant difference in relative abundance between DNA and RNA, mainly with pH, temperature, TAN, free ammonia, conductivity and Na^+^ (Table 1, Additional file 1: Figure S5). Almost no significant correlations between bacterial OTUs and total VFA or acetate were observed, in contrast to the archaea.

**Table 1:** Overview of the number of bacterial OTUs that show a significant (*P* < 0.05) correlation with the operational parameters, based on the Kendall rank correlation coefficient. Only bacterial OTUs with a significant (*P* < 0.05) difference between DNA and RNA (203 OTUs in total) were considered

Parameter	DNA	RNA
pH	5	7
Temperature	44	29
TAN	40	62
Free ammonia	8	16
Conductivity	35	43
Na^+^	41	7
K^+^	0	0
Total VFA	1	2
Acetate	0	0
Propionate	0	0
Butyrate	0	0
SRT	1	0

*TAN* total ammonia nitrogen, *VFA* volatile fatty acids, *SRT* sludge retention time

**Fig. 5:**
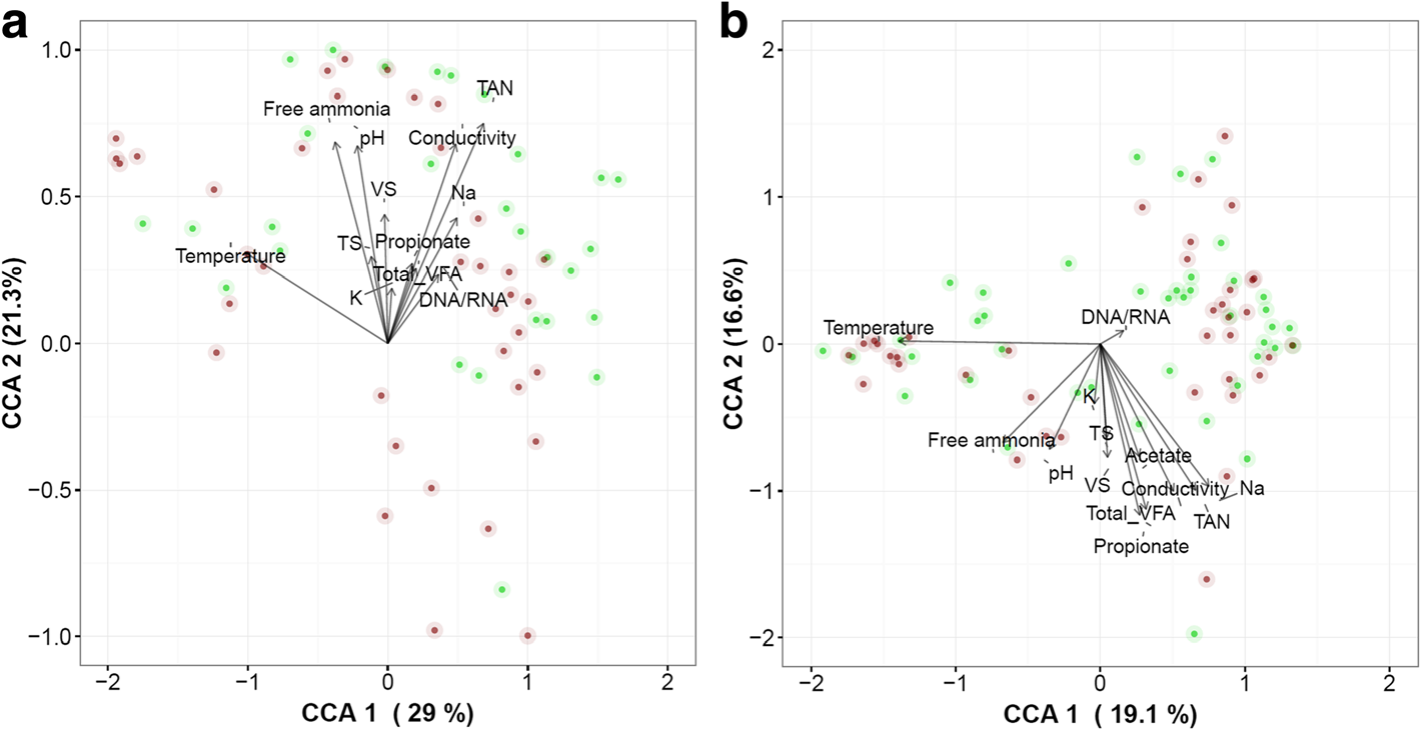
Canonical correspondence analysis of the **a** archaeal and **b** bacterial community, including the DNA (red) and RNA (green) profiles of each sample at OTU level. PERMANOVA was carried out to evaluate the effect of operational parameters on community composition, and significant (*P* < 0.01) correlations are presented by the arrows

The overall archaeal community was primarily shaped by temperature, pH, TAN, free ammonia, conductivity, VS and TS (*P* = 0.001). The Na^+^ (*P* = 0.006), K^+^ (*P* = 0.002), propionate (*P* = 0.003) and total VFA (*P* = 0.002) also had a strong impact on the archaeal community (Fig. 5). A similar observation was made for the bacterial community, with temperature, pH, TAN, free ammonia, conductivity, Na^+^, K^+^, VS and propionate (*P* < 0.001) as main factors, with a strong effect of TS (*P* = 0.006), Mg^2+^ (*P* = 0.010), acetate (*P* = 0.005), butyrate (*P* = 0.005) and total VFA (*P* = 0.007) as well. The significant (*P* < 0.001) difference between DNA and RNA profiles, as observed based on beta diversity analysis, was confirmed for the archaeal and bacterial community.

### Co-occurrence and subcommunities between bacteria and archaea

The combined co-occurrence profile of bacteria and archaea contained only OTUs that correlated (*P* < 0.001) with at least one other OTU, which resulted in 128 (93.4%) archaeal and 639 (80.4%) bacterial OTUs on DNA level and 82 (59.9%) archaeal and 698 (87.8%) bacterial OTUs on RNA level. In total, 6311 and 6391 significant correlations were observed on DNA and RNA levels, respectively, of which 1414 were present both in the DNA and RNA co-occurrence profiles. On the DNA level, one central subcommunity was identified, containing 251 OTUs, alongside four other subcommunities with 128, 101, 95 and 82 OTUs, respectively (Additional file 1: Figure S6). The RNA co-occurrence profile contained five major subcommunities of 188, 166, 124, 124 and 77 OTUs, respectively (Additional file 1: Figure S7). The network statistics Degree, Betweenness, Closeness and Eigenvector centrality did not entail the identification of specific keystone species at the OTU level, due to the presence of multiple different OTUs in the network. At the genus level, however, the archaeon Candidatus *Methanomethylophilus* held a central role at the RNA level based on Betweenness and Degree statistics. In contrast, the bacterium *Fastidiosipila* held a more central role in the network at the DNA level, also based on Betweenness and Degree statistics.

A significantly (*P* < 0.001) higher average closeness centrality was detected in the co-occurrence profile on the OTU level of both the bacterial and archaeal communities at the RNA level compared with the DNA level (Table 2). The archaeal and bacterial community significantly differed on RNA level in terms of average degree centrality (*P* = 0.0010) and on DNA level in terms of average betweenness centrality (*P* = 0.049).

**Table 2:** Overview of the average network statistics Degree, Betweenness, Closeness, and Eigenvector centrality, determined separately for the bacterial and archaeal community and the DNA and RNA profiles. Significant (*P* < 0.05) differences, as determined by a *t* test, between bacteria and archaea or between DNA and RNA are indicated by different letters (a–d)

Centrality parameter	DNA		RNA	
	Bacteria	Archaea	Bacteria	Archaea
Degree	15.9 ± 15.3	19.1 ± 19.5	15.7 ± 13.1^a^	22.0 ± 16.0^a^
Betweenness	1095 ± 1507^b^	1473 ± 2048^b^	1138 ± 1581	1271 ± 1786
Closeness	8.8 × 10^−5^ ± 8.1 × 10^−5c^	8.8 × 10^−5^ ± 8.1 × 10^−5d^	1.2 × 10^− 4^ ± 1.1 × 10^−4c^	1.2 × 10^−4^ ± 1.1 × 10^−4d^
Eigenvector	0.034 ± 0.167	0.054 ± 0.197	0.035 ± 0.159	0.046 ± 0.196

### Functional prediction based on 16S rRNA (gene) data

An estimation of the metabolic potential of the archaea and bacteria, both at the DNA and RNA levels, based on the KEGG pathway database resulted in the identification of 3067 and 6404 KO numbers for archaea and bacteria, respectively. The average KO number per sample was significantly higher for DNA than for RNA for archaea (*P* < 0.0001), while this was not the case for bacteria (*P* = 0.30) (Table 3). For the archaea, 986 KO numbers had a significantly (*P* < 0.001) different relative abundance between DNA and RNA, while for the bacteria, 3155 significantly different KO numbers were identified. A separate KEGG-based visualization of the main pathways by bacteria and archaea in the AD process indicated similar coverage of the metabolic pathways on DNA and RNA levels, which was especially the case for methanogenesis in the general methane metabolism (Additional file 1: Figure S8).

**Table 3:** Overview of the KO number statistics

	DNA		RNA	
	Bacteria	Archaea	Bacteria	Archaea
Average KO numbers	5721 ± 546	2927 ± 152^a^	5839 ± 564	2511 ± 330^a^
Significant higher KO numbers	663	630	2522	338

Significant (*P* < 0.001) differences, as determined by a *t* test, between bacteria and archaea or between DNA and RNA are indicated by letter “^a^”. Significant higher KO numbers were determined based on a significant higher relative abundance on DNA or RNA level

## Discussion

A clear difference between the total (DNA) and active (RNA) microbial community was detected at the individual OTU level as well as alpha and beta diversity of the bacterial and archaeal communities. Nonetheless, these differences were significantly more pronounced for archaea as compared with bacteria. Though similar operational parameters affected the overall archaeal and bacterial community, at the OTU level, a clear difference was detected for the DNA and RNA data response between bacteria and archaea. Both co-occurrence and functional prediction profiles also showed a clear difference between DNA and RNA, but this did not affect metabolic pathway structure prediction.

### The archaeal active community profile reflects specialization and organization

Microbial community diversity has been postulated to reflect functional stability in AD and other similar ecosystems in several studies, whether or not taking only richness or evenness into account [62–65]. However, this apparent relation was not observed in other studies [9, 10, 66], which leads one to question to which extent functionality really depends on diversity [67]. In our study, a significantly lower alpha diversity was observed for the active compared with the total archaeal community, mainly through the difference in richness. This contrast indicates a high degree of functional specialization, despite the high metabolic potential through a high archaeal diversity. Lin et al. [68] observed a clear centralization of functionality for methanogenesis, based on functional pathway prediction, despite a high alpha diversity. This was also observed in our study, as a significantly lower KO number (based on the KEGG database) was observed at the RNA compared with the DNA level for archaea, indicating a predicted higher degree of functional specialization. This relates with the fact that, in general, only two major pathways are responsible for methane production in AD, i.e., hydrogenotrophic and acetoclastic methanogenesis, which do not require a diverse archaeal community. Most digesters in our study were dominated by hydrogenotrophic methanogens, both on DNA and RNA levels, and this points to an even higher degree of functional specialization. The high archaeal diversity at the DNA compared with the RNA level can be considered a pool of ‘reserve players’ that are not active but are able to take over when digester conditions change, related to the susceptibility and narrow optimal operational parameter range of most methanogens [20, 69]. Overall, the clear differentiation between the DNA and the RNA profiles, based on alpha and beta diversity measures but related with operational data, reflects a well-organized methanogenic community.

### The active and total bacterial community have a similar structure but different composition

The differentiation between the DNA and the RNA profiles in terms of alpha diversity that was observed for the archaeal community was not observed for the bacterial community. This indicates a similar structural organization of the total and active bacterial community. Beta diversity analysis, however, revealed a significant differentiation between the total and active community, which shows a difference in community composition. The high degree of variance between DNA and RNA based on the unweighted UniFrac measure confirms that the presence/absence of different OTUs and not their relative abundance is responsible for the difference between the bacterial DNA and RNA profiles [70], yet this strongly depends on sequencing depth, which was in this case similar for the RNA and DNA data.

The similarity of the structural organization of the bacterial community on DNA and RNA level is the consequence of the inherent different involvement and properties of the bacterial and archaeal community in the AD process. While archaea only have to perform two methanogenic pathways in AD, the bacterial community carries out numerous pathways, which requires a higher active community diversity. This is also reflected in the higher total KO number for the bacterial compared with the archaeal community. The bacterial community, in general, has a higher resilience [71] in contrast with the sensitivity of the methanogenic archaea [72, 73], which coincides with a more diverse active bacterial community. In our study, almost all archaeal OTUs with a significant difference in relative abundance between DNA and RNA levels have a significant negative correlation with VFA on the RNA level, which confirms their sensitivity, and this is not observed for the bacterial OTUs. The on average higher growth rate of bacteria compared with archaea, especially acetoclastic methanogens [73, 74], is another important factor that emphasizes the active bacterial community, relative to the active archaeal community, especially following disturbances.

The difference in community composition between the total and active bacterial communities can be considered the result of two factors. First, multiple bacterial species are able to occupy the same niche, as is the case for syntrophic acetate oxidation [75] and glucose fermentation [76]. Second, often the same species is able to engage in multiple pathways, such as *Clostridium kluyveri* that can perform chain elongation on ethanol and VFA [77, 78].

The importance of deterministic factors to shape the microbial community composition has been demonstrated extensively [79, 80], and also, in our study, the significant effect of several operational factors, including temperature, pH and (free) ammonia, on the total and active microbial community was confirmed. However, the similar bacterial community structure (alpha diversity) but different community composition (beta diversity) on DNA and RNA level indicates that stochastic processes play a major role in determining which of the bacterial species actually become actively involved in the AD process [81].

### The future of 16S rRNA (gene) analysis: does combination of DNA and RNA profile provide a broad overview for more detailed analysis?

Microbial community analysis via the 16S rRNA gene is a common practice and serves as the basis for several molecular techniques. In this research, this was supplemented with the analysis of the 16S rRNA gene transcripts to make an estimation if there is a difference between the active and total microbial community, which was unambiguously confirmed with our results. The difference between the DNA and the RNA profiles, however, also has a potential temporal and spatial character. First, there is a high degree of natural community variation, even at stable conditions, in AD [5, 8, 82], mainly related to biological interactions [83], as observed in the co-occurrence parameters. Second, shifts in the key operational parameters, i.e., pH and (free) ammonia, related to the feedstock composition [84], may provoke a diverging effect between the DNA and the RNA community profiles [85]. Third, spatial variation, related to mixing or heating [86], might also lead to a divergence in the DNA and RNA community profiles, which may affect β-diversity [87]. Hence, considering the natural temporal variations, a divergence between the DNA and the RNA community profiles, related to for example operational parameter variation, could be used as a *proxy* of ‘real’ community stability. The suitability of the 16S rRNA (gene) to characterize mixed microbial communities is, however, becoming more and more questionable. This is related to the difference in 16S rRNA gene copy numbers between species, yet this also accounts for other (marker) genes [88, 89]. Normalization of the 16S rRNA gene copy number was applied for the Tax4Fun analysis, as this is inherent to this software package [47]. However, no additional normalization to the copy number was applied, given the high variation in 16S rRNA gene copy number between species, which can vary from one to 15 [90], and the fact that species level classification was not possible, due to the limited amplicon length. The ‘omics’ techniques have been postulated as possessing a higher resolution and sensitivity [91] and also suffer from other issues, as mentioned earlier.

The strength of the combined analysis of the microbial community based on the 16S rRNA and its transcripts, in our opinion, is related to potential to gain a quick and broad overview changes not only in the total and active community, but also even into potential collaboration and competition between micro-organisms and overall functionality.

Co-occurrence analysis of the combined archaeal and bacterial community revealed a globally similar pattern between DNA and RNA, related to the comparable amount of correlations and organization in subcommunities. On the OTU level, however, there is a clear differentiation between the DNA and the RNA profiles that is related to the difference in bacterial and archaeal community composition, as determined by beta diversity analysis. Co-occurrence analysis is a suitable tool to estimate potential interactions between micro-organisms [54, 92]. In this study, Candidatus *Methanomethylophilus* was identified as a potential keystone species in the active community, based on the co-occurrence network statistics. In line with the co-occurrence analysis, pathway prediction via the KEGG database could be used to make an estimation of the metabolic potential of the microbial community. However, functional prediction based on the 16S rRNA gene should be considered with great care, given (1) the fact that taxonomic identification does not necessarily relate with the presence of functional genes and (2) the high degree of variation in the 16S rRNA gene copy number between species [90]. In this study, the specialization of the active archaeal community compared with the total archaeal community was confirmed with the pathway prediction results, which is in line with the results of Lin et al. [68].

Hence, a 16S rRNA (gene)-based approach can provide a broad overview of community presence, activity and potential performance, which could serve as a valuable overview and basis to engage in more detailed community profiling via alternative techniques.

## Conclusions

Alpha and beta diversity, co-occurrence and functional prediction profiles indicated an increased level of specialization in the active archaeal community. In contrast, the total and active bacterial community showed a similar community structure; however, community composition also more strongly differed between the total and active communities. The clear difference between RNA- and DNA-based community screening confirms the importance of this combined approach to obtain a broad general overview, not only on the total and active community, but also in terms of potential collaboration and competition and predicted functionality. These results then serve as a basis for further integrated process engineering of the anaerobic digestion process.

## Additional files

Additional file 1: Supporting Information. This file contains all the supporting information that is related to the manuscript, including additional results, figure captions and tables. This file is to be published online as Supporting Information. The figures are included in separate files and labeled Figures S1–S8. (ZIP 20215 kb)

Additional file 2: OTU table archaea. This file contains the OTU table of the archaeal community that was generated as described in the ‘Methods’ section and that was used for further analyses and creation of some of the figures. This file is to be published online as Supporting Information. (CSV 88 kb)

Additional file 3: OTU table bacteria. This file contains the OTU table of the bacterial community that was generated as described in the ‘Methods’ section and that was used for further analyses and creation of some of the figures. This file is to be published online as Supporting Information. (CSV 568 kb)
