# Supplementary material for: The active microbial community more accurately reflects the anaerobic digestion process: 16S rRNA (gene) sequencing as a predictive tool
Source: Microbiome. 2018 Apr 2;6:63. doi: 10.1186/s40168-018-0449-9 (PMC5879801; doi:10.1186/s40168-018-0449-9)
Supplement: Supplementary file 1 — Supporting Information. This file contains all the supporting information that is related to the manuscript, including additional results, figure captions and tables. This file is to be published online as Supporting Information. The figures are included in separate files and labeled Figures S1–S8. (ZIP 20215 kb) [file 40168_2018_449_MOESM1_ESM.zip › 20161012_DNAvsRNAinADPaper_FigureS5.pdf]

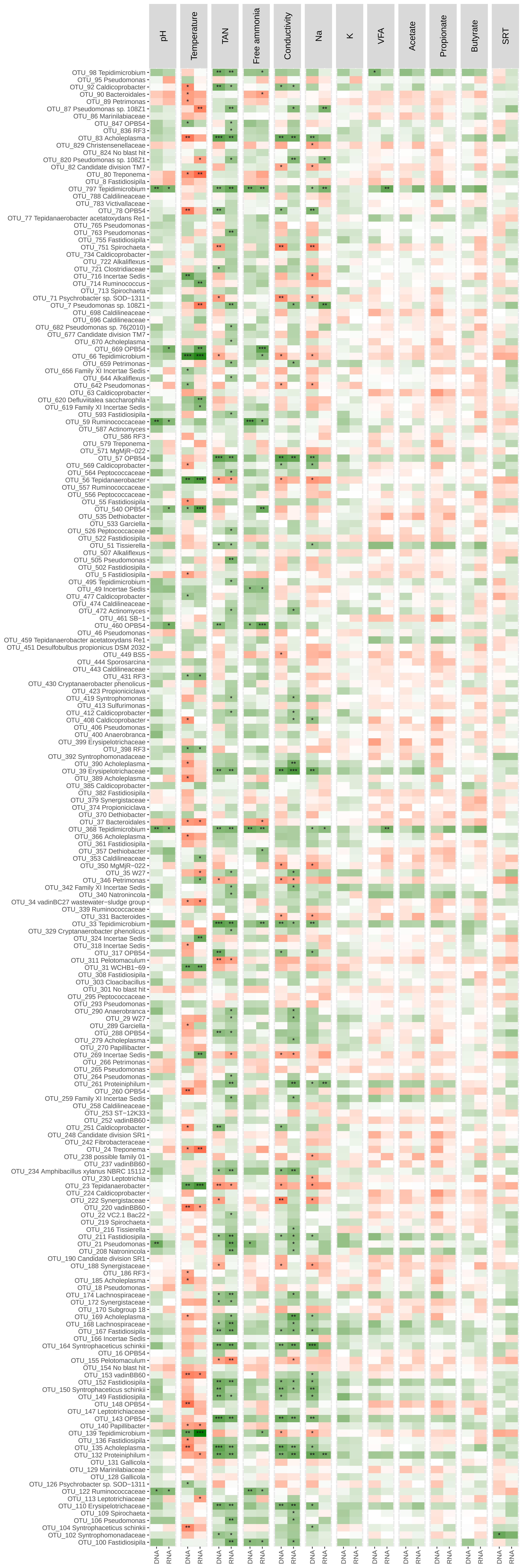

pH
Temperature
TAN
Free ammonia
Conductivity
Na
K
VFA
Acetate
Propionate
Butyrate
SRT
OTU_98 Tepidimicrobium
OTU_95 Pseudomonas
OTU_92 Caldicoprobacter
OTU_90 Bacteroidales
OTU_89 Petrimonas
OTU_87 Pseudomonas sp. 108Z1
OTU_86 Marinilabiaceae
OTU_847 OPB54
OTU_836 RF3
OTU_83 Acholeplasma
OTU_829 Christensenellaceae
OTU_824 No blast hit
OTU_820 Pseudomonas sp. 108Z1
OTU_82 Candidate division TM7
OTU_80 Treponema
OTU_8 Fastidiosipila
OTU_797 Tepidimicrobium
OTU_788 Caldilineaceae
OTU_783 Victivallaceae
OTU_78 OPB54
OTU_77 Tepidanaerobacter acetatoxydans Re1
OTU_765 Pseudomonas
OTU_763 Pseudomonas
OTU_755 Fastidiosipila
OTU_751 Spirochaeta
OTU_734 Caldicoprobacter
OTU_722 Alkaliflexus
OTU_721 Clostridiaceae
OTU_716 Incertae Sedis
OTU_714 Ruminococcus
OTU_713 Spirochaeta
OTU_71 Psychrobacter sp. SOD−1311
OTU_7 Pseudomonas sp. 108Z1
OTU_698 Caldilineaceae
OTU_696 Caldilineaceae
OTU_682 Pseudomonas sp. 76(2010)
OTU_677 Candidate division TM7
OTU_670 Acholeplasma
OTU_669 OPB54
OTU_66 Tepidimicrobium
OTU_659 Petrimonas
OTU_656 Family XI Incertae Sedis
OTU_644 Alkaliflexus
OTU_642 Pseudomonas
OTU_63 Caldicoprobacter
OTU_620 Defluviitalea saccharophila
OTU_619 Family XI Incertae Sedis
OTU_593 Fastidiosipila
OTU_59 Ruminococcaceae
OTU_587 Actinomyces
OTU_586 RF3
OTU_579 Treponema
OTU_571 MgMjR−022
OTU_57 OPB54
OTU_569 Caldicoprobacter
OTU_564 Peptococcaceae
OTU_56 Tepidanaerobacter
OTU_557 Ruminococcaceae
OTU_556 Peptococcaceae
OTU_55 Fastidiosipila
OTU_540 OPB54
OTU_535 Dethiobacter
OTU_533 Garciella
OTU_526 Peptococcaceae
OTU_522 Fastidiosipila
OTU_51 Tissierella
OTU_507 Alkaliflexus
OTU_505 Pseudomonas
OTU_502 Fastidiosipila
OTU_5 Fastidiosipila
OTU_495 Tepidimicrobium
OTU_49 Incertae Sedis
OTU_477 Caldicoprobacter
OTU_474 Caldilineaceae
OTU_472 Actinomyces
OTU_461 SB−1
OTU_460 OPB54
OTU_46 Pseudomonas
OTU_459 Tepidanaerobacter acetatoxydans Re1
OTU_451 Desulfobulbus propionicus DSM 2032
OTU_449 BS5
OTU_444 Sporosarcina
OTU_443 Caldilineaceae
OTU_431 RF3
OTU_430 Cryptanaerobacter phenolicus
OTU_423 Propioniciclava
OTU_419 Syntrophomonas
OTU_413 Sulfurimonas
OTU_412 Caldicoprobacter
OTU_408 Caldicoprobacter
OTU_406 Pseudomonas
OTU_400 Anaerobranca
OTU_399 Erysipelotrichaceae
OTU_398 RF3
OTU_392 Syntrophomonadaceae
OTU_390 Acholeplasma
OTU_39 Erysipelotrichaceae
OTU_389 Acholeplasma
OTU_385 Caldicoprobacter
OTU_382 Fastidiosipila
OTU_379 Synergistaceae
OTU_374 Propioniciclava
OTU_370 Dethiobacter
OTU_37 Bacteroidales
OTU_368 Tepidimicrobium
OTU_366 Acholeplasma
OTU_361 Fastidiosipila
OTU_357 Dethiobacter
OTU_353 Caldilineaceae
OTU_350 MgMjR−022
OTU_35 W27
OTU_346 Petrimonas
OTU_342 Family XI Incertae Sedis
OTU_340 Natronincola
OTU_34 vadinBC27 wastewater−sludge group
OTU_339 Ruminococcaceae
OTU_331 Bacteroides
OTU_33 Tepidimicrobium
OTU_329 Cryptanaerobacter phenolicus
OTU_324 Incertae Sedis
OTU_318 Incertae Sedis
OTU_317 OPB54
OTU_311 Pelotomaculum
OTU_31 WCHB1−69
OTU_308 Fastidiosipila
OTU_303 Cloacibacillus
OTU_301 No blast hit
OTU_295 Peptococcaceae
OTU_293 Pseudomonas
OTU_290 Anaerobranca
OTU_29 W27
OTU_289 Garciella
OTU_288 OPB54
OTU_279 Acholeplasma
OTU_270 Papillibacter
OTU_269 Incertae Sedis
OTU_266 Petrimonas
OTU_265 Pseudomonas
OTU_264 Pseudomonas
OTU_261 Proteiniphilum
OTU_260 OPB54
OTU_259 Family XI Incertae Sedis
OTU_258 Caldilineaceae
OTU_253 ST−12K33
OTU_252 vadinBB60
OTU_251 Caldicoprobacter
OTU_248 Candidate division SR1
OTU_242 Fibrobacteraceae
OTU_24 Treponema
OTU_238 possible family 01
OTU_237 vadinBB60
OTU_234 Amphibacillus xylanus NBRC 15112
OTU_230 Leptotrichia
OTU_23 Tepidanaerobacter
OTU_224 Caldicoprobacter
OTU_222 Synergistaceae
OTU_220 vadinBB60
OTU_22 VC2.1 Bac22
OTU_219 Spirochaeta
OTU_216 Tissierella
OTU_211 Fastidiosipila
OTU_21 Pseudomonas
OTU_208 Natronincola
OTU_190 Candidate division SR1
OTU_188 Synergistaceae
OTU_186 RF3
OTU_185 Acholeplasma
OTU_18 Pseudomonas
OTU_174 Lachnospiraceae
OTU_172 Synergistaceae
OTU_170 Subgroup 18
OTU_169 Acholeplasma
OTU_168 Lachnospiraceae
OTU_167 Fastidiosipila
OTU_166 Incertae Sedis
OTU_164 Syntrophaceticus schinkii
OTU_16 OPB54
OTU_155 Pelotomaculum
OTU_154 No blast hit
OTU_153 vadinBB60
OTU_152 Fastidiosipila
OTU_150 Syntrophaceticus schinkii
OTU_149 Fastidiosipila
OTU_148 OPB54
OTU_147 Leptotrichiaceae
OTU_143 OPB54
OTU_140 Papillibacter
OTU_139 Tepidimicrobium
OTU_136 Fastidiosipila
OTU_135 Acholeplasma
OTU_132 Proteiniphilum
OTU_131 Gallicola
OTU_129 Marinilabiaceae
OTU_128 Gallicola
OTU_126 Psychrobacter sp. SOD−1311
OTU_122 Ruminococcaceae
OTU_113 Leptotrichiaceae
OTU_110 Erysipelotrichaceae
OTU_109 Spirochaeta
OTU_106 Pseudomonas
OTU_104 Syntrophaceticus schinkii
OTU_102 Syntrophomonadaceae
OTU_100 Fastidiosipila
DNA
RNA
