# Supplementary material for: The active microbial community more accurately reflects the anaerobic digestion process: 16S rRNA (gene) sequencing as a predictive tool
Source: Microbiome. 2018 Apr 2;6:63. doi: 10.1186/s40168-018-0449-9 (PMC5879801; doi:10.1186/s40168-018-0449-9)
Supplement: Supplementary file 1 — Supporting Information. This file contains all the supporting information that is related to the manuscript, including additional results, figure captions and tables. This file is to be published online as Supporting Information. The figures are included in separate files and labeled Figures S1–S8. (ZIP 20215 kb) [file 40168_2018_449_MOESM1_ESM.zip › 20161104_DNAvsRNAinADPaperSI.docx]

**Supporting Information**

Title: The active microbial community more accurately reflects the anaerobic digestion process: 16S rRNA (gene) sequencing as a predictive tool

**Jo De Vrieze^1,2^, Ameet J. Pinto^3^, William T. Sloan^2^, Umer Z. Ijaz^2^***

^1^Center for Microbial Ecology and Technology (CMET), Ghent University, Coupure Links 653, B-9000 Gent, Belgium

^2^Infrastructure and Environment Research Division, School of Engineering, University of Glasgow, UK

^3^Northeastern University, 360 Huntington Avenue, Boston, MA 02115, USA

^🖂^ Correspondence to: Umer Zeeshan Ijaz, Rankine Building, School of Engineering, University of Glasgow, Oakfield Avenue, Glasgow G12 8LT, UK, +44(0)141-330-6458, Umer.Ijaz@glasgow.ac.ukContents

[Contents 2](#_Toc464059502)

[S1. Amplicon sequencing primer sets and PCR protocol 3](#_Toc464059503)

[S2. Volatile fatty acid analysis 4](#_Toc464059504)

[S3. Overview of the operational data 5](#_Toc464059505)

[S4. Overview figure legends 11](#_Toc464059506)

[S5. References 13](#_Toc464059507)

# S1. Amplicon sequencing primer sets and PCR protocol

The PCR mix of in total 20 µL MyTaq buffer contained 50 pg of (c)DNA, 15 pmol of each primer (Table S1), 1.5 units of MyTaq DNA polymerase (Bioline London, UK) and 2 µL of BioStabII PCR optimizer (Sigma-Aldrich, St. Louis, MO). The PCR program consisted of a predenaturation step of 2 min at 96 °C, followed by 20 cycles of 15 s at 96 °C, 30 s at 50°C and 90 s at 70 °C. The 341F and 785R primer set was used for the bacteria in a single PCR run, while a nested PCR approach with the primer sets 340F and 1000R in the first, and 341F and 806R in the second run was used to amplify archaea. After the first run containing 20 cycles, 1 µL of PCR product was used as template for the second run that was identical to the first run, apart from the different primer set. A separate and nested approach was used for the archaea to ensure sufficient coverage of the archaeal community.

**Table S1** Overview of the primer sets used for amplicon sequencing of the DNA and cDNA samples.

| Primer | Sequence | Reference |
| --- | --- | --- |
| 341F | 5’- NNNNNNNNNTCCTACGGGNGGCWGCAG | ([Klindworth et al 2013](#_ENREF_2)) |
| 785R | 5’- NNNNNNNNNNTGACTACHVGGGTATCTAAKCC | ([Klindworth et al 2013](#_ENREF_2)) |
| 340F | 5’- CCCTAYGGGGYGCASCAG | ([Gantner et al 2011](#_ENREF_1)) |
| 1000R | 5’- GAGARGWRGTGCATGGCC | ([Gantner et al 2011](#_ENREF_1)) |
| 806R | 5’- GGACTACNNGGGTATCTAAT | ([Sundberg et al 2013](#_ENREF_3)) |

# S2. Volatile fatty acid analysis

The different VFA concentrations (C2-C8) were measured using gas chromatography (GC-2014, Shimadzu®, The Netherlands), equipped with a DB-FFAP 123-3232 column (30 m x 0.32 mm x 0.25 µm; Agilent, Belgium) and a flame ionization detector (FID). Samples were conditioned with sulphuric acid and sodium chloride, and 2-methyl hexanoic acid was used as internal standard for quantification of the extraction with diethyl ether. The sample (1 µL) was injected at 200 ºC with a split ratio of 60 and a purge flow of 3 mL min^-1^. The oven temperature increased by 6 ºC min^-1^ from 110 ºC to 165 ºC, where it was kept for 2 min. The FID had a temperature of 220 ºC. The carrier gas was N_2_ at a flow rate of 2.49 mL min^-1^. The detection limit was 30 mg L^-1^ for acetate, 10 mg L^-1^ for propionate and 2 mg L^-1^ for the other VFA.

# S3. Overview of the operational data

**Table S2** Overview of the operational parameters in the samples of the 48 full-scale anaerobic digestion plants. TAN = total ammonia, VFA = volatile fatty acids, SRT = sludge retention time, VS = volatile solids, TS = total solids, CSTR = continuously stirred tank reactor, DRANCO = dry anaerobic composting, NA = data not available, BDL = below detection limit.

| **Name** | **Type** | **SRT** | **Temperature** | **pH** | **TAN** | **Free ammonia** | **TS** | **VS** |
| --- | --- | --- | --- | --- | --- | --- | --- | --- |
|  |  | d | °C |  | mg N L^-1^ | mg N L^-1^ | g L^-1^ | g L^-1^ |
|  |  |  |  |  |  |  |  |  |
| OWSg | DRANCO | 22 | 50 | 8.23 | 2390 | 798 | 231.0 | 127.4 |
| OWSd | CSTR | NA | 50 | 8.44 | 3264 | 1464 | 95.3 | 74.4 |
| OWSc | CSTR | NA | 50 | 8.47 | 3358 | 1563 | 94.4 | 73.7 |
| OWSe | DRANCO | NA | 50 | 8.51 | 3245 | 1585 | 93.0 | 72.7 |
| OWSf | DRANCO | 20 | 50 | 7.98 | 2151 | 473 | 188.7 | 103.4 |
| OWSa | DRANCO | 850 | 35 | 8.07 | 5961 | 701 | 325.8 | 144.7 |
| OWSb | DRANCO | 34 | 37 | 8.03 | 2107 | 257 | 406.5 | 106.3 |
| OWSi | DRANCO | 58 | 35 | 8.38 | 4007 | 857 | 272.4 | 106.4 |
| OWSj | DRANCO | 67 | 47 | 8.29 | 4636 | 1501 | 310.5 | 126.6 |
| OWSl | DRANCO | 33 | 47 | 8.43 | 3371 | 1341 | 378.1 | 121.9 |
| OWSk | DRANCO | 21 | 48 | 8.21 | 2267 | 674 | 327.0 | 153.7 |
| OWSm | DRANCO | 31 | 45 | 8.38 | 3509 | 1200 | 372.0 | 157.5 |
| BTd | CSTR | 40 | 35 | 7.61 | 1488 | 66 | 85.9 | 62.2 |
| BTa | CSTR | 42 | 43.5 | 8.21 | 3349 | 811 | 190.7 | 69.6 |
| BTb | CSTR | 35 | 38 | 7.82 | 3699 | 310 | 115.7 | 83.2 |
| BTc | CSTR | 47 | 46 | 8.08 | 3844 | 835 | 99.7 | 76.7 |
| Bte | CSTR | 35 | 38 | 7.69 | 2251 | 143 | 78.2 | 54.4 |
| MCFR | CSTR | 33 | 37 | 7.6 | 1663 | 82 | 126.6 | 117.0 |
| VS | CSTR | 40 | 55 | 7.6 | 1199 | 164 | 43.1 | 31.3 |
| GGPA | CSTR | 40 | 37 | 7.7 | 2669 | 163 | 104.4 | 51.4 |
| QEFA | CSTR | 45 | 37 | 7.96 | 4681 | 495 | 118.0 | 68.9 |
| StVA | CSTR | 80 | 37 | 7.34 | 3567 | 98 | 147.1 | 90.3 |
| BBA | CSTR | 36 | 54 | 7.87 | 2966 | 646 | 53.2 | 39.5 |
| RF | CSTR | 40 | 53 | 7.39 | 605 | 48 | 23.2 | 15.1 |
| BIOEA | CSTR | 40 | 53 | 7.65 | 2021 | 276 | 48.1 | 27.1 |
| AgroEA | CSTR | 40 | 37 | 8.04 | 5971 | 743 | 130.8 | 80.5 |
| WEEA | CSTR | 60 | 37 | 7.87 | 4114 | 360 | 101.8 | 66.9 |
| APA | CSTR | 40 | 53 | 7.74 | 2540 | 413 | 74.3 | 42.8 |
| SENA | CSTR | 40 | 34 | 7.69 | 3702 | 183 | 112.4 | 61.2 |
| AgroGA | CSTR | 40 | 37 | 7.87 | 3736 | 327 | 123.4 | 70.5 |
| ODBA | CSTR | 45 | 37 | 7.74 | 3929 | 261 | 131.1 | 67.7 |
| CAZ | CSTR | 40 | 37 | 7.7 | 2753 | 168 | 135.6 | 82.5 |
| BioBA | CSTR | 40 | 37 | 8.01 | 4201 | 492 | 147.1 | 81.6 |
| VCEA | CSTR | 40 | 34 | 8.09 | 3877 | 448 | 171.8 | 101.7 |
| ABRA | CSTR | 40 | 37 | 7.34 | 3625 | 100 | 144.1 | 100.0 |
| WatIep | CSTR | 40 | 37 | 7.91 | 3387 | 323 | 100.2 | 71.1 |
| AVECO | CSTR | 40 | 37 | 7.97 | 3829 | 413 | 139.3 | 93.5 |
| VHA | CSTR | 40 | 37 | 7.52 | 3831 | 158 | 113.1 | 40.3 |
| Myd | CSTR | 40 | 37 | 7.57 | 811 | 37 | 19.9 | 12.5 |
| BFB | CSTR | 40 | 38 | 8.18 | 5301 | 918 | 108.8 | 70.1 |
| Bio7A | CSTR | 40 | 37 | 8.36 | 4869 | 1114 | 157.8 | 102.6 |
| ENG | CSTR | 40 | 53 | 7.87 | 1772 | 368 | 90.4 | 71.4 |
| GEA | CSTR | 35 | 38 | 8.18 | 4538 | 786 | 138.6 | 88.5 |
| GEB | CSTR | 35 | 38 | 8.09 | 4496 | 654 | 153.6 | 102.2 |
| BoBA | CSTR | 40 | 37 | 8.13 | 4451 | 662 | 111.0 | 68.9 |
| KAA | CSTR | 45 | 37 | 8.09 | 3568 | 490 | 136.8 | 67.5 |
| KAD | CSTR | 45 | 37 | 8.13 | 3707 | 551 | 131.5 | 61.0 |
| BCIA | CSTR | 80 | 34 | 8.13 | 3735 | 467 | 124.2 | 80.6 |

| **Name** | **Conductivity** | **Na** | **K** | **Ca** | **Mg** | **Total VFA** | **Aceate** |
| --- | --- | --- | --- | --- | --- | --- | --- |
|  | mS cm^-1^ | g L^-1^ | g L^-1^ | g L^-1^ | g L^-1^ | mg COD L^-1^ | mg COD L^-1^ |
|  |  |  |  |  |  |  |  |
| OWSg | 14.7 | 1.87 | 3.40 | 0.46 | BDL | 489 | 397 |
| OWSd | 31.5 | 0.12 | 5.57 | BDL | BDL | 241 | 241 |
| OWSc | 33.5 | 0.22 | 5.07 | 0.20 | BDL | 303 | 303 |
| OWSe | 32.1 | 0.27 | 5.52 | 0.20 | 0.10 | 297 | 297 |
| OWSf | 26.2 | 1.30 | 2.71 | 0.50 | 0.08 | 953 | 625 |
| OWSa | 64.6 | 5.01 | 3.73 | 0.49 | BDL | 27496 | 5728 |
| OWSb | 34.5 | 2.59 | 6.89 | 0.11 | 0.13 | 830 | 830 |
| OWSi | 29.5 | 2.23 | 3.16 | 0.03 | BDL | 10464 | 1263 |
| OWSj | 27.7 | 2.91 | 3.01 | BDL | BDL | 620 | 442 |
| OWSl | 14.32 | 3.51 | 3.36 | 0.14 | BDL | 112 | BDL |
| OWSk | 22.65 | 2.70 | 2.60 | BDL | BDL | 588 | BDL |
| OWSm | 23.25 | 3.09 | 2.58 | 0.26 | 0.08 | 831 | 337 |
| BTd | 11.4 | 1.06 | 3.06 | BDL | BDL | 536 | 194 |
| BTa | 30 | 1.74 | 3.40 | BDL | BDL | 944 | 766 |
| BTb | 35.1 | 1.58 | 3.92 | BDL | 0.20 | 1723 | 1063 |
| BTc | 34.4 | 1.06 | 3.79 | 0.30 | BDL | 2782 | 2151 |
| Bte | 12.73 | 0.67 | 3.26 | 0.45 | BDL | 462 | 169 |
| MCFR | 10.3 | 0.95 | 0.59 | 0.36 | 0.08 | 167 | BDL |
| VS | 13.65 | 0.79 | 1.78 | 0.40 | 0.08 | 483 | 483 |
| GGPA | 21.6 | 1.29 | 2.38 | 0.54 | BDL | 255 | 104 |
| QEFA | 59.3 | 8.80 | 4.87 | 0.34 | BDL | 11556 | 3586 |
| StVA | 23.8 | 0.99 | 2.78 | 0.36 | 0.08 | 12297 | 3590 |
| BBA | 29.1 | 1.05 | 0.89 | BDL | BDL | 1550 | 770 |
| RF | 16.11 | 0.76 | 2.93 | 0.36 | 0.18 | 172 | 157 |
| BIOEA | 29.9 | 2.42 | 2.82 | BDL | 0.15 | 389 | 302 |
| AgroEA | 48.5 | 2.75 | 3.33 | 0.34 | 0.14 | 1239 | 546 |
| WEEA | 33.6 | 1.82 | 2.80 | 0.30 | 0.10 | 658 | 507 |
| APA | 26.4 | 1.46 | 2.24 | 0.35 | BDL | 1748 | 1233 |
| SENA | 42.4 | 4.58 | 1.79 | 0.33 | BDL | 7455 | 1069 |
| AgroGA | 35.4 | 3.65 | 2.47 | BDL | BDL | 524 | 441 |
| ODBA | 36.3 | 3.92 | 1.84 | 0.26 | BDL | 244 | 138 |
| CAZ | 29.2 | 4.89 | 3.06 | 0.23 | BDL | 7285 | 1355 |
| BioBA | 39.1 | 3.28 | 3.31 | 0.19 | BDL | 987 | 502 |
| VCEA | 31 | 4.46 | 2.58 | 0.16 | BDL | 345 | 251 |
| ABRA | 23.4 | 1.36 | 3.92 | 0.32 | 0.07 | 644 | 549 |
| WatIep | 25.7 | 2.17 | 2.74 | 0.42 | 0.22 | 398 | 223 |
| AVECO | 27.4 | 3.83 | 4.04 | 0.36 | 0.23 | 2464 | 776 |
| VHA | 33.6 | 1.72 | 1.77 | 0.44 | 0.09 | 6312 | 3893 |
| Myd | 12.98 | 0.23 | 2.19 | BDL | 0.10 | 312 | 167 |
| BFB | 41.2 | 2.76 | 5.23 | 0.34 | 0.14 | 364 | 191 |
| Bio7A | 35.7 | 1.27 | 4.94 | 0.20 | 0.11 | 764 | 307 |
| ENG | 21.4 | 0.18 | 3.99 | 0.38 | 0.22 | 406 | 367 |
| GEA | 43.7 | 7.92 | 3.61 | BDL | 0.12 | 14138 | 987 |
| GEB | 39.1 | 7.63 | 3.54 | BDL | BDL | 24452 | 2205 |
| BoBA | 40.2 | 3.38 | 3.71 | 0.16 | 0.08 | 428 | 379 |
| KAA | 42.2 | 6.41 | 2.79 | 0.15 | BDL | 335 | 335 |
| KAD | 40.7 | 6.50 | 2.74 | 0.14 | BDL | 81 | 81 |
| BCIA | 32.5 | 3.99 | 3.45 | BDL | BDL | 391 | 270 |

| **Name** | **Propionate** | **Isobutyrate** | **Butyrate** | **Isovalerate** | **Valerate** | **Isocaproate** |  |
| --- | --- | --- | --- | --- | --- | --- | --- |
|  | mg COD L^-1^ | mg COD L^-1^ | mg COD L^-1^ | mg COD L^-1^ | mg COD L^-1^ | mg COD L^-1^ |  |
|  |  |  |  |  |  |  |  |
| OWSg | 64 | BDL | BDL | 29 | BDL | BDL |  |
| OWSd | BDL | BDL | BDL | BDL | BDL | BDL |  |
| OWSc | BDL | BDL | BDL | BDL | BDL | BDL |  |
| OWSe | BDL | BDL | BDL | BDL | BDL | BDL |  |
| OWSf | 188 | 58 | BDL | 82 | BDL | BDL |  |
| OWSa | 15982 | 1182 | 527 | 2855 | 1056 | 40 |  |
| OWSb | BDL | BDL | BDL | BDL | BDL | BDL |  |
| OWSi | 8739 | 189 | 33 | 196 | BDL | 44 |  |
| OWSj | 133 | BDL | BDL | 45 | BDL | BDL |  |
| OWSl | BDL | BDL | 51 | 61 | BDL | BDL |  |
| OWSk | 348 | BDL | 109 | 130 | BDL | BDL |  |
| OWSm | 345 | 55 | BDL | 94 | BDL | BDL |  |
| BTd | 342 | BDL | BDL | BDL | BDL | BDL |  |
| BTa | 121 | BDL | BDL | 57 | BDL | BDL |  |
| BTb | 333 | 91 | BDL | 237 | BDL | BDL |  |
| BTc | 379 | 65 | 73 | 114 | BDL | BDL |  |
| Bte | 294 | BDL | BDL | BDL | BDL | BDL |  |
| MCFR | 167 | BDL | BDL | BDL | BDL | BDL |  |
| VS | BDL | BDL | BDL | BDL | BDL | BDL |  |
| GGPA | 62 | 21 | BDL | 68 | BDL | BDL |  |
| QEFA | 7368 | 196 | 141 | 265 | BDL | BDL |  |
| StVA | 2498 | 795 | 2102 | 1022 | 1286 | 126 |  |
| BBA | 631 | 61 | BDL | 87 | BDL | BDL |  |
| RF | BDL | BDL | BDL | 14 | BDL | BDL |  |
| BIOEA | 87 | BDL | BDL | BDL | BDL | BDL |  |
| AgroEA | 398 | 68 | 82 | 144 | BDL | BDL |  |
| WEEA | BDL | BDL | BDL | 151 | BDL | BDL |  |
| APA | 432 | BDL | BDL | 83 | BDL | BDL |  |
| SENA | 6065 | 109 | BDL | 212 | BDL | BDL |  |
| AgroGA | BDL | BDL | BDL | 83 | BDL | BDL |  |
| ODBA | 38 | 17 | BDL | 50 | BDL | BDL |  |
| CAZ | 5385 | 153 | BDL | 392 | BDL | BDL |  |
| BioBA | 413 | BDL | BDL | 72 | BDL | BDL |  |
| VCEA | 47 | BDL | BDL | 46 | BDL | BDL |  |
| ABRA | 95 | BDL | BDL | BDL | BDL | BDL |  |
| WatIep | 71 | 29 | 16 | 60 | BDL | BDL |  |
| AVECO | 1125 | 154 | BDL | 410 | BDL | BDL |  |
| VHA | 1227 | 326 | 279 | 586 | BDL | BDL |  |
| Myd | 20 | 49 | BDL | 76 | BDL | BDL |  |
| BFB | 74 | 16 | BDL | 83 | BDL | BDL |  |
| Bio7A | 399 | BDL | 58 | BDL | BDL | BDL |  |
| ENG | BDL | BDL | BDL | 39 | BDL | BDL |  |
| GEA | 12471 | 158 | BDL | 522 | BDL | BDL |  |
| GEB | 18943 | 744 | BDL | 2360 | 94 | 106 |  |
| BoBA | BDL | BDL | BDL | 48 | BDL | BDL |  |
| KAA | BDL | BDL | BDL | BDL | BDL | BDL |  |
| KAD | BDL | BDL | BDL | BDL | BDL | BDL |  |
| BCIA | 64 | BDL | BDL | 56 | BDL | BDL |  |

| **Name** | **Caproate** | **Heptanoate** | **Octanoate** |  |
| --- | --- | --- | --- | --- |
|  | mg COD L^-1^ | mg COD L^-1^ | mg COD L^-1^ |  |
|  |  |  |  |  |
| OWSg | BDL | BDL | BDL |  |
| OWSd | BDL | BDL | BDL |  |
| OWSc | BDL | BDL | BDL |  |
| OWSe | BDL | BDL | BDL |  |
| OWSf | BDL | BDL | BDL |  |
| OWSa | 71 | BDL | 57 |  |
| OWSb | BDL | BDL | BDL |  |
| OWSi | BDL | BDL | BDL |  |
| OWSj | BDL | BDL | BDL |  |
| OWSl | BDL | BDL | BDL |  |
| OWSk | BDL | BDL | BDL |  |
| OWSm | BDL | BDL | BDL |  |
| BTd | BDL | BDL | BDL |  |
| BTa | BDL | BDL | BDL |  |
| BTb | BDL | BDL | BDL |  |
| BTc | BDL | BDL | BDL |  |
| Bte | BDL | BDL | BDL |  |
| MCFR | BDL | BDL | BDL |  |
| VS | BDL | BDL | BDL |  |
| GGPA | BDL | BDL | BDL |  |
| QEFA | BDL | BDL | BDL |  |
| StVA | 718 | 161 | BDL |  |
| BBA | BDL | BDL | BDL |  |
| RF | BDL | BDL | BDL |  |
| BIOEA | BDL | BDL | BDL |  |
| AgroEA | BDL | BDL | BDL |  |
| WEEA | BDL | BDL | BDL |  |
| APA | BDL | BDL | BDL |  |
| SENA | BDL | BDL | BDL |  |
| AgroGA | BDL | BDL | BDL |  |
| ODBA | BDL | BDL | BDL |  |
| CAZ | BDL | BDL | BDL |  |
| BioBA | BDL | BDL | BDL |  |
| VCEA | BDL | BDL | BDL |  |
| ABRA | BDL | BDL | BDL |  |
| WatIep | BDL | BDL | BDL |  |
| AVECO | BDL | BDL | BDL |  |
| VHA | BDL | BDL | BDL |  |
| Myd | BDL | BDL | BDL |  |
| BFB | BDL | BDL | BDL |  |
| Bio7A | BDL | BDL | BDL |  |
| ENG | BDL | BDL | BDL |  |
| GEA | BDL | BDL | BDL |  |
| GEB | BDL | BDL | BDL |  |
| BoBA | BDL | BDL | BDL |  |
| KAA | BDL | BDL | BDL |  |
| KAD | BDL | BDL | BDL |  |
| BCIA | BDL | BDL | BDL |  |

# S4. Overview figure legends

**Figure S1** Boxplots representing the distribution of the relative abundance of the 58 archaeal OTUs that had a significant (*P* < 0.05) difference between DNA (red) and RNA (green).

**Figure S2a** Boxplots representing the distribution of the relative abundance of the first 50 bacterial OTUs out of 203 that had a significant (*P* < 0.05) difference between DNA (red) and RNA (green).

**Figure S2b** Boxplots representing the distribution of the relative abundance of the second 50 bacterial OTUs out of 203 that had a significant (*P* < 0.05) difference between DNA (red) and RNA (green).

**Figure S2c** Boxplots representing the distribution of the relative abundance of the third 50 bacterial OTUs out of 203 that had a significant (*P* < 0.05) difference between DNA (red) and RNA (green).

**Figure S2d** Boxplots representing the distribution of the relative abundance of the last 54 bacterial OTUs out of 203 that had a significant (*P* < 0.05) difference between DNA (red) and RNA (green).

**Figure S3** Non-metric distance scaling (NMDS) analysis of the Bray-Curtis dissimilarity index of the (a) archaeal and (b) bacterial community and the weighed Unifrac distance of the (c) archaeal and (d) bacterial community at OTU level. The DNA (red) and RNA (green) based community profiles of the same samples were connected by means of a grey line. The circles represent the 95% value of the standard error of the average value of the DNA (red) and RNA (green) indices.

**Figure S4** Variance between the DNA (red) and RNA (green) profile of the different samples, determined as the distance from the centroid (average value) of the principal coordinates of the Bray-Curtis dissimilarity index the (a) archaeal and (b) bacterial community, the unweighed Unifrac distance of the (c) archaeal and (d) bacterial community, and the weighed Unifrac distance of the (e) archaeal and (f) bacterial community at OTU level. Significant differences in variance between the archaeal and bacterial community were determine by means of a t-test. Significant values are marked with * (*P* < 0.05), ** (*P* < 0.01), and *** (*P* < 0.001).

Figure S5 Correlation pattern of the 203 bacterial OTUs that had a significant (*P* < 0.05) difference in relative abundance between DNA and RNA with the main operational parameters, based on the Kendall rank correlation coefficient. Significant positive (green) or negative (red) correlations are marked with * (*P* < 0.05), ** (*P* < 0.01), or *** (*P* < 0.001). TAN= total ammonia nitrogen, VFA = volatile fatty acids, SRT = sludge retention time.

**Figure S6** Co-occurrence profile on DNA level including those OTUs with a significant (P < 0.001) correlation with at least one other OTU, as determined by the Spearman's rank correlation coefficient. Different colours represent different subcommunities, which are clusters of OTUs with a significant correlation ρ > 0.5 amongst each other.

**Figure S7** Co-occurrence profile on RNA level including those OTUs with a significant (P < 0.001) correlation with at least one other OTU, as determined by the Spearman's rank correlation coefficient. Different colours represent different subcommunities, which are clusters of OTUs with a significant correlation ρ > 0.5 amongst each other.

**Figure S8** KEGG based visualization of the methane metabolism pathway (ko00680). Functions with predicted upregulated transcripts are shown in red, while non-regulated transcripts are shown in grey. The intensity of the red colour relates with the square root transformed relative abundance of the specific transcript. The left side of the box of each transcript represents the RNA based prediction and the right side of each box represents the DNA based prediction.

# S5. References

Gantner S, Andersson AF, Alonso-Saez L, Bertilsson S (2011). Novel primers for 16S rRNA-based archaeal community analyses in environmental samples. *J Microbiol Methods* **84:** 12-18.

Klindworth A, Pruesse E, Schweer T, Peplies J, Quast C, Horn M *et al* (2013). Evaluation of general 16S ribosomal RNA gene PCR primers for classical and next-generation sequencing-based diversity studies. *Nucleic Acids Research* **41:** 11.

Sundberg C, Al-Soud WA, Larsson M, Alm E, Yekta SS, Svensson BH *et al* (2013). 454 pyrosequencing analyses of bacterial and archaeal richness in 21 full-scale biogas digesters. *FEMS Microbiol Ecol* **85:** 612-626.
