# Supplementary figures and images for: The active microbial community more accurately reflects the anaerobic digestion process: 16S rRNA (gene) sequencing as a predictive tool
Source: Microbiome. 2018 Apr 2;6:63. doi: 10.1186/s40168-018-0449-9 (PMC5879801; doi:10.1186/s40168-018-0449-9)

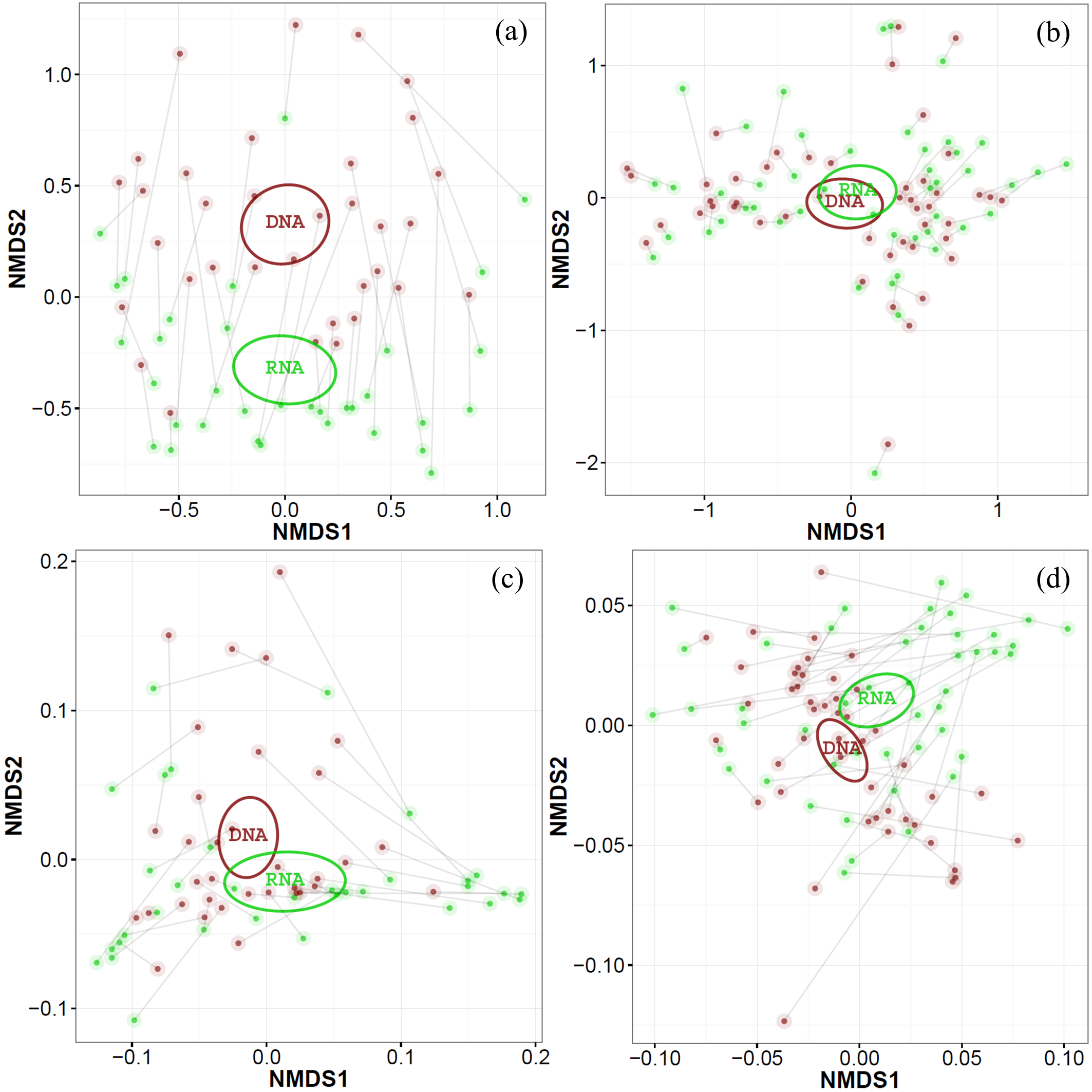

Supplement: Supplementary file 1 — Supporting Information. This file contains all the supporting information that is related to the manuscript, including additional results, figure captions and tables. This file is to be published online as Supporting Information. The figures are included in separate files and labeled Figures S1–S8. (ZIP 20215 kb) [file 40168_2018_449_MOESM1_ESM.zip › 20161012_DNAvsRNAinADPaper_FigureS3.tif]

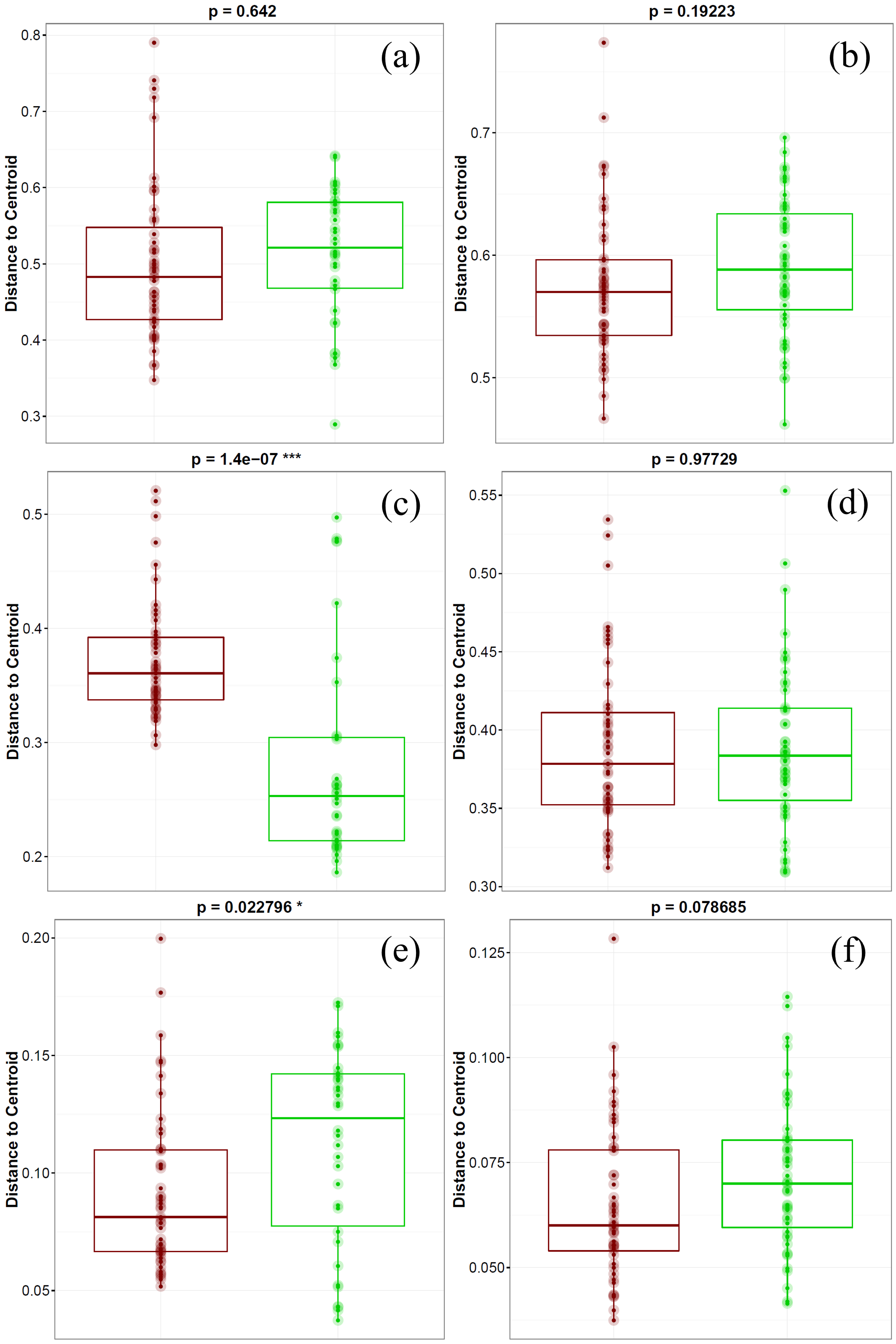

Supplement: Supplementary file 1 — Supporting Information. This file contains all the supporting information that is related to the manuscript, including additional results, figure captions and tables. This file is to be published online as Supporting Information. The figures are included in separate files and labeled Figures S1–S8. (ZIP 20215 kb) [file 40168_2018_449_MOESM1_ESM.zip › 20161012_DNAvsRNAinADPaper_FigureS4.tif]

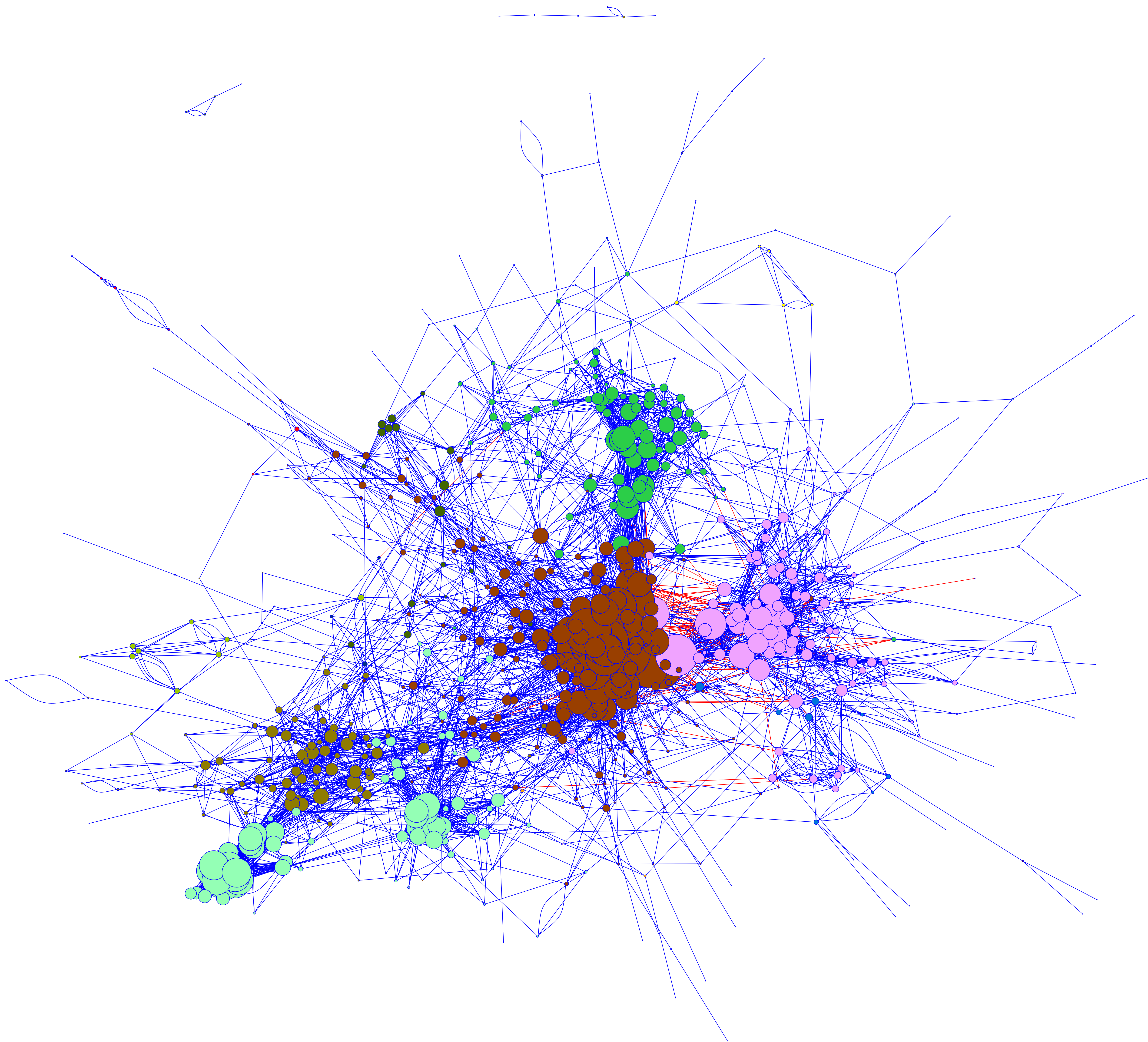

Supplement: Supplementary file 1 — Supporting Information. This file contains all the supporting information that is related to the manuscript, including additional results, figure captions and tables. This file is to be published online as Supporting Information. The figures are included in separate files and labeled Figures S1–S8. (ZIP 20215 kb) [file 40168_2018_449_MOESM1_ESM.zip › 20161012_DNAvsRNAinADPaper_FigureS6.pdf]

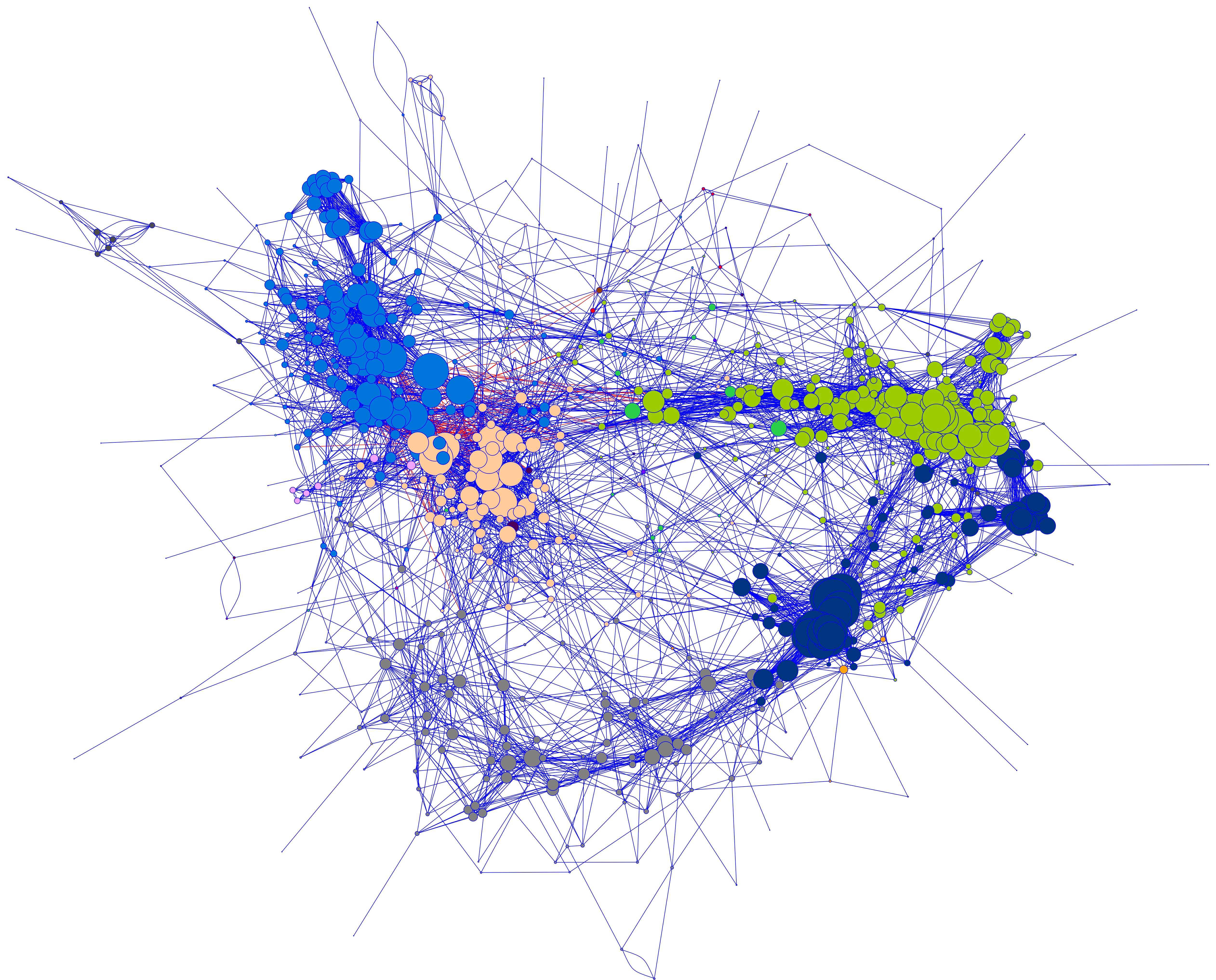

Supplement: Supplementary file 1 — Supporting Information. This file contains all the supporting information that is related to the manuscript, including additional results, figure captions and tables. This file is to be published online as Supporting Information. The figures are included in separate files and labeled Figures S1–S8. (ZIP 20215 kb) [file 40168_2018_449_MOESM1_ESM.zip › 20161012_DNAvsRNAinADPaper_FigureS7.pdf]

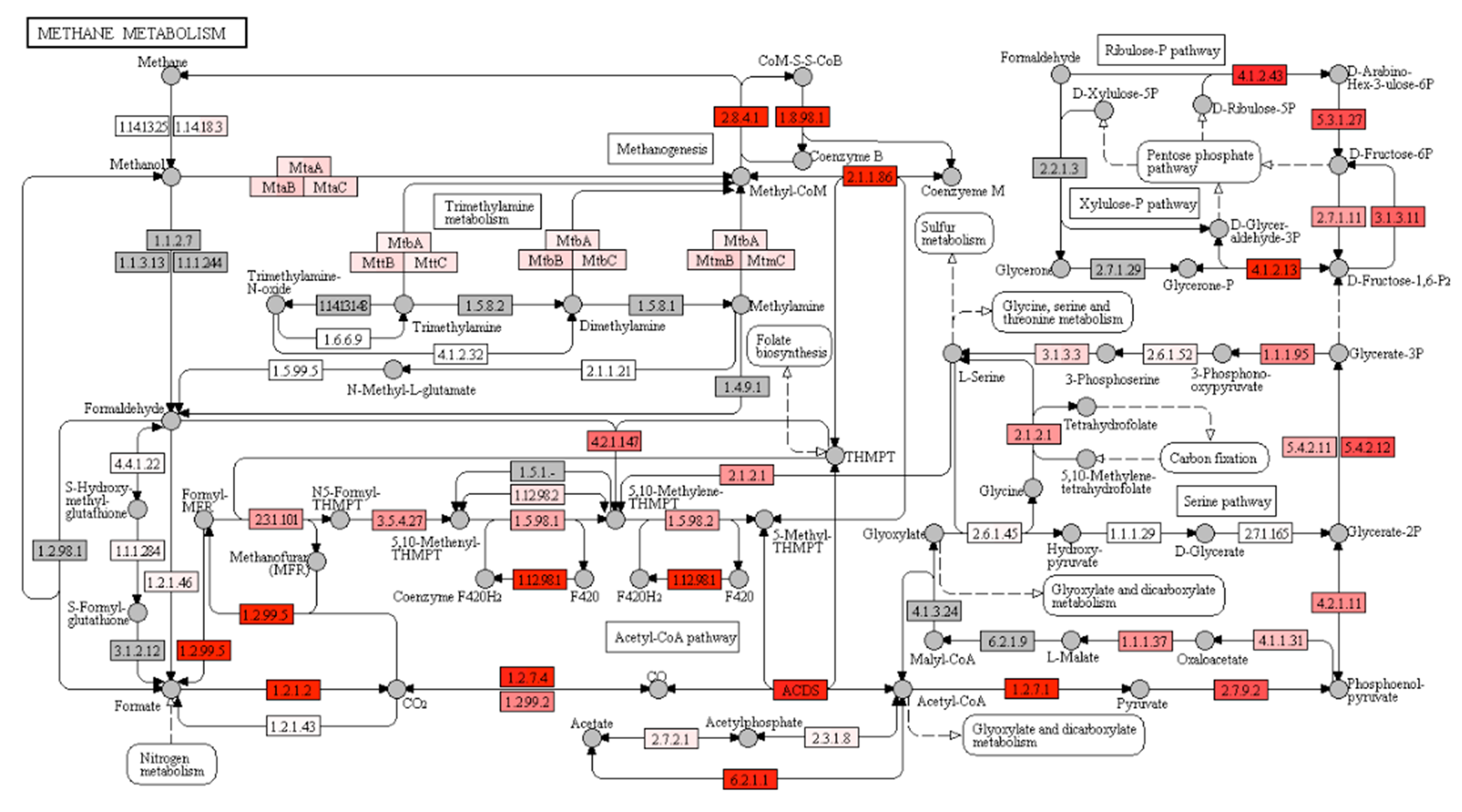

Supplement: Supplementary file 1 — Supporting Information. This file contains all the supporting information that is related to the manuscript, including additional results, figure captions and tables. This file is to be published online as Supporting Information. The figures are included in separate files and labeled Figures S1–S8. (ZIP 20215 kb) [file 40168_2018_449_MOESM1_ESM.zip › 20161012_DNAvsRNAinADPaper_FigureS8.tif]
